# Corrigendum: The genetic landscape of benign thyroid nodules revealed by whole exome and transcriptome sequencing

**DOI:** 10.1038/ncomms16129

**Published:** 2017-07-04

**Authors:** Lei Ye, Xiaoyi Zhou, Fengjiao Huang, Weixi Wang, Yicheng Qi, Heng Xu, Yang Shu, Liyun Shen, Xiaochun Fei, Jing Xie, Min Cao, Yulin Zhou, Wei Zhu, Shu Wang, Guang Ning, Weiqing Wang

Nature Communications 8: Article number:15533 ; DOI: 10.1038/ncomms15533 (2017); Published 06 05 2017; Updated 07 04 2017

The original version of this Article contained an error in the formatting of the author name Yang Shu, which was incorrectly given as Shu Yang. This has now been corrected in both the PDF and HTML versions of the Article.

