# Supplementary material for: The genetic landscape of benign thyroid nodules revealed by whole exome and transcriptome sequencing
Source: Nat Commun. 2017 Jun 5;8:15533. doi: 10.1038/ncomms15533 (PMC5465355; doi:10.1038/ncomms15533)
Supplement: Supplementary Information — Supplementary Figures and Supplementary Tables [file ncomms15533-s11.pdf]

Supplementary Figure 1

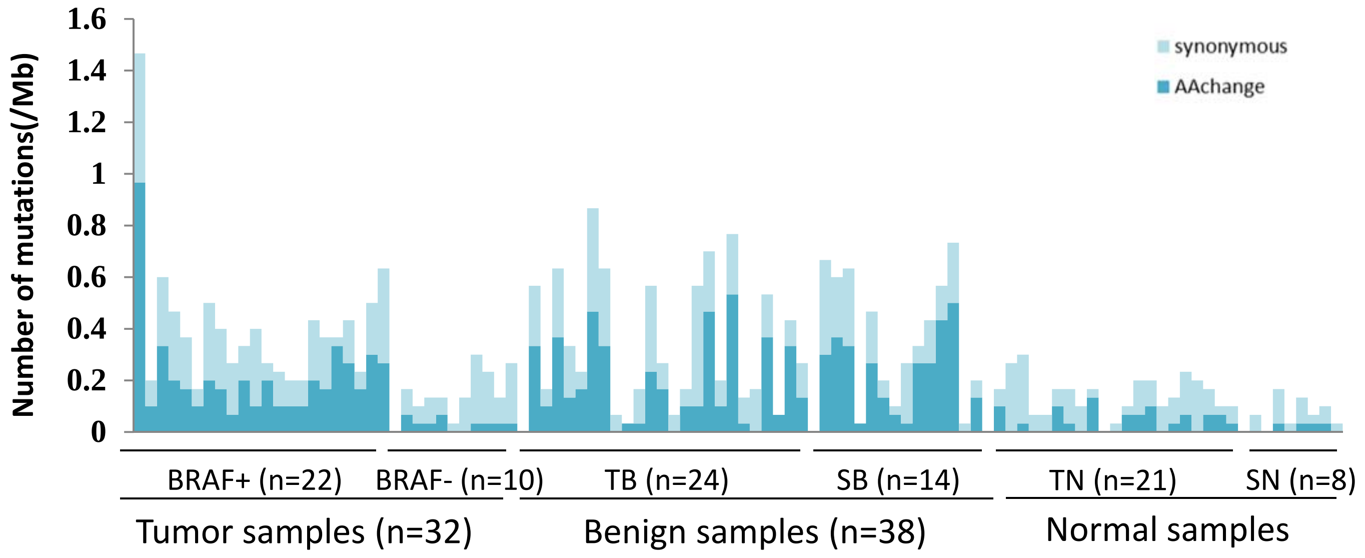

Supplementary Figure 1 The density of somatic mutations in 99 samples. Mutations density in exonic region (per Mb) of each sample in this cohort. AA changes included all missense SNV, stop gain SNV and indels. Tumor samples were divided in the group with and that without BRAF^{V600E}.

Supplementary Figure 2

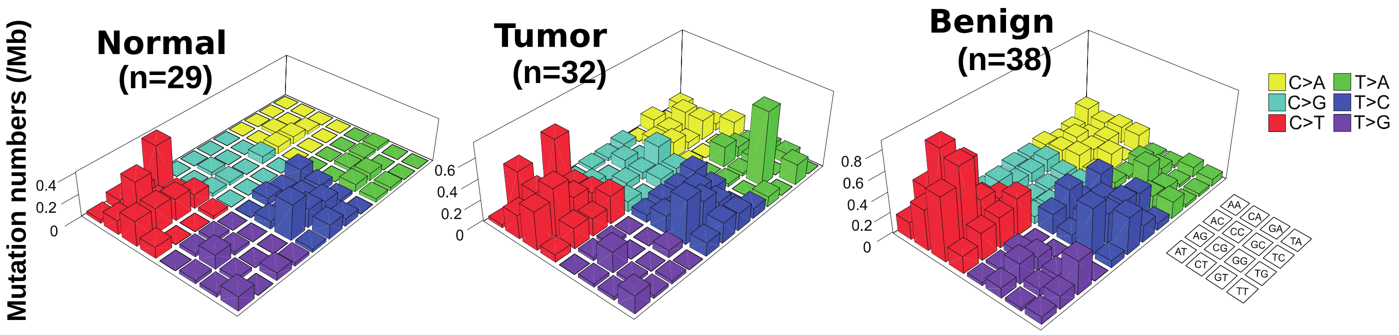

Supplementary Figure 2 Mean frequency of mutational context.
Six types of base substitutions were further divided into 16 categories according to the flanking nucleotides. The pattern of C>T transition was enriched in PTC, especially at TCA and TCC trinucleotides. In benign nodules, despite of the presence of a medium enrichment of C>T, the T>C transition context presented as the most common pattern.

Supplementary Figure 3

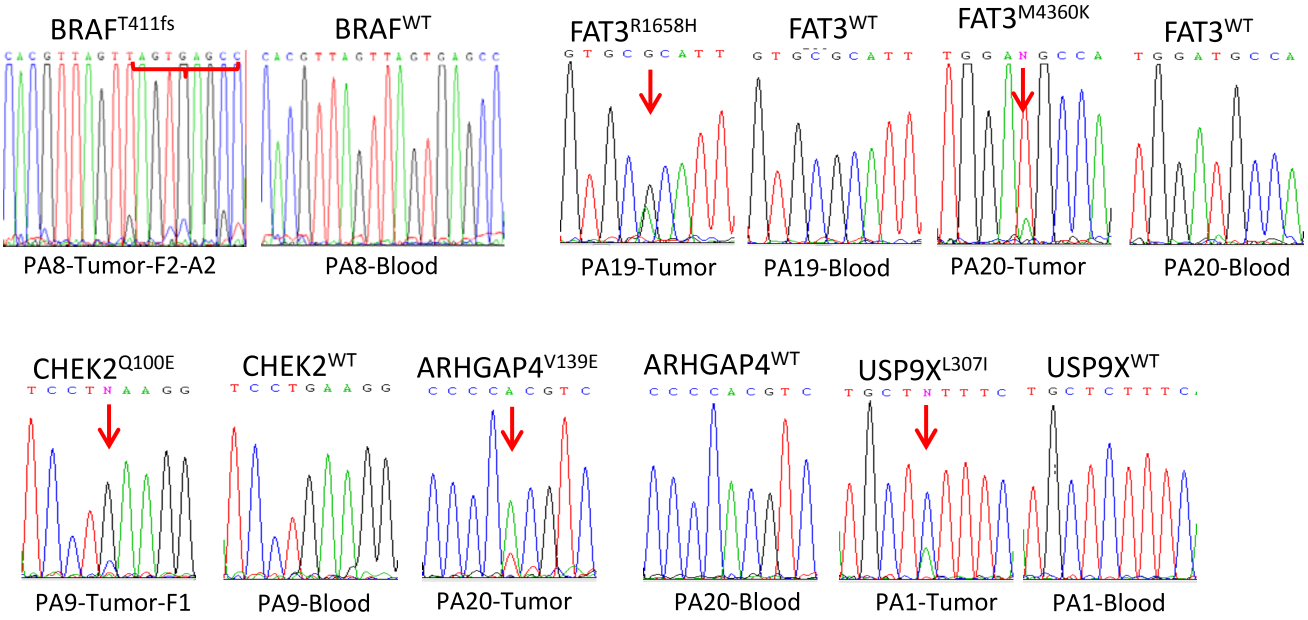

Supplementary Figure 3

Validation of putative functional somatic mutations in our cohort

PCR and sanger sequencing were performed to validate somatic mutations presented in at least two PTC samples.

Supplementary Figure 4

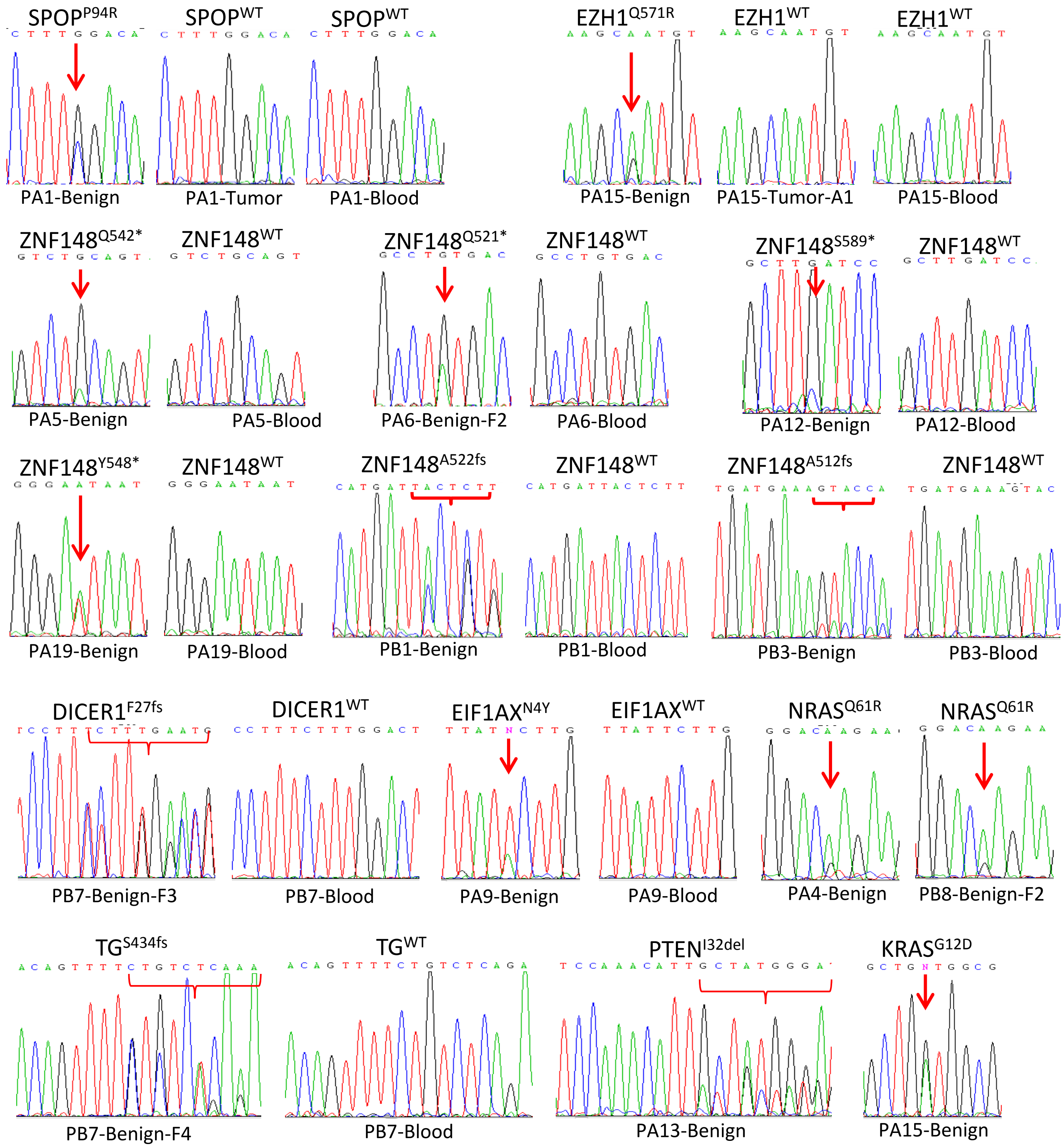

Supplementary Figure 4

Validation of putative functional somatic mutations in our cohort

PCR and sanger sequencing were performed to validate somatic mutations presented in at least two BTN.

Supplementary Figure 5

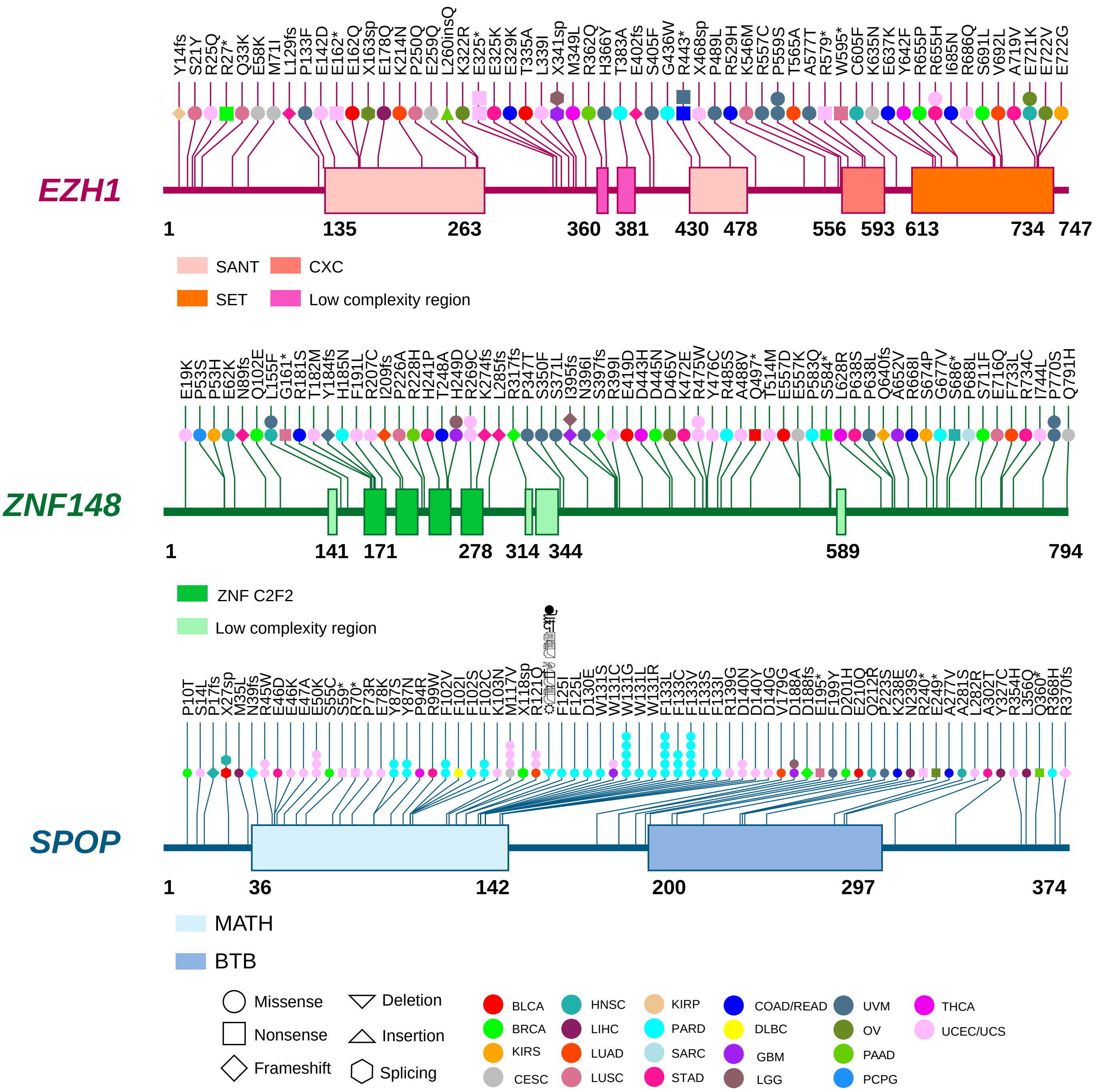

Supplementary Figure 5

Schematic representation of mutations in SPOP, EZH1 and ZNF148 in TCGA database.

Supplementary Figure 6

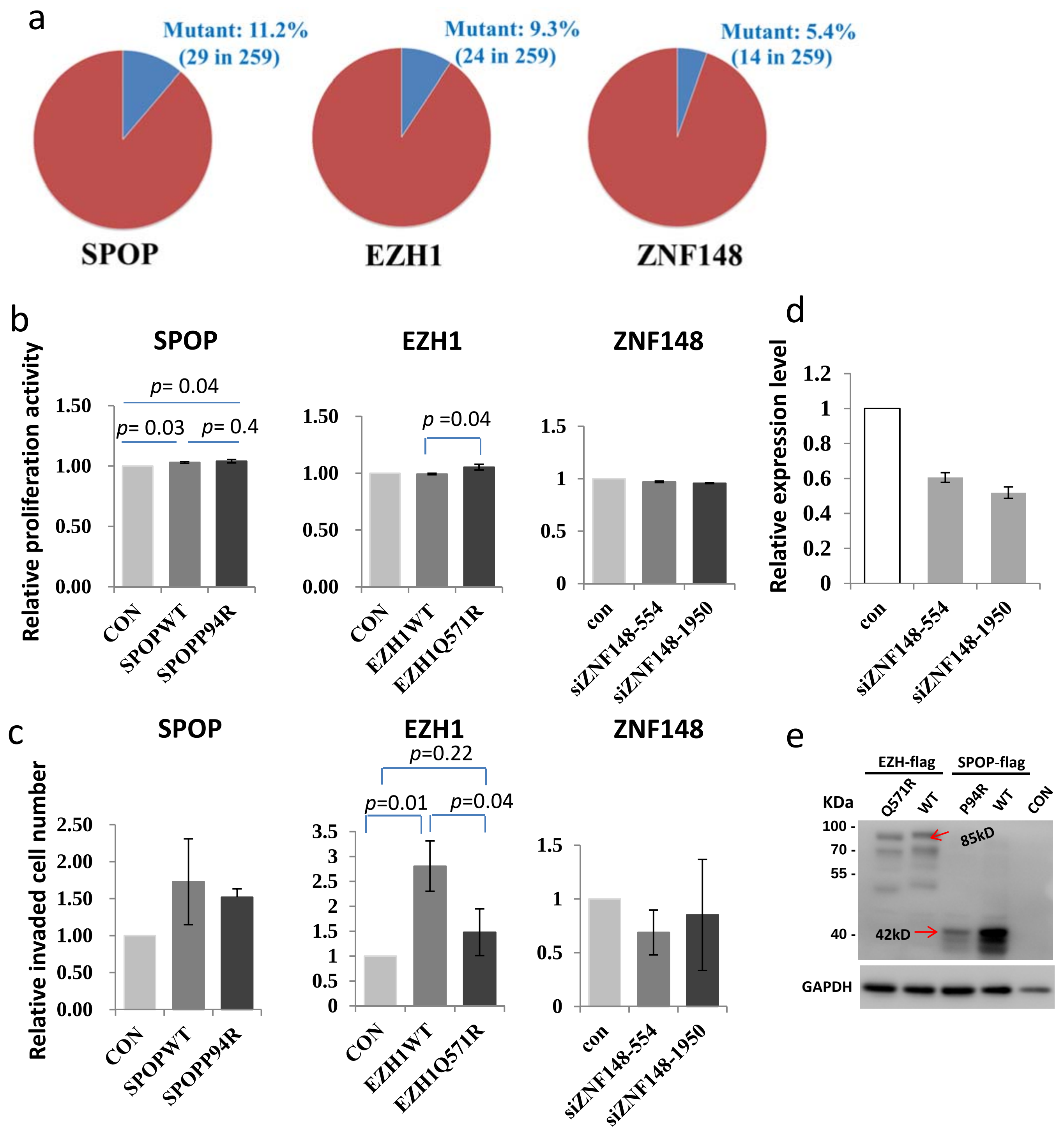

Supplementary Figure 6 Functional analysis of three mutated genes in BTNs.

(a) Frequencies of the three most common recurrent mutations, $SPOP^{P94R}$, EZH^{Q571R} and ZNF148 in BTNs in a cohort of 259 patients.

(b) CCK8 assay for proliferation of NThy cells which were transfected with relevant plasmid or siRNA. (c) Transwell assay for invaded cells.

(d) The efficiency of siRNA transfection. siZNF148-554 and 1905, two individual lines of ZNF148 RNAi. Data were presented as mean $\pm$ SD (n = 3).

(e) The protein level of SPOP or EZH1 in transfected NThy cells.

CON, control; $SPOP^{WT}$, wild type SPOP protein; $SPOP^{P94R}$, SPOP protein with p.P94R mutation; $EZH1^{WT}$, wild type EZH1 protein; $EZH1^{Q571R}$, EZH1 protein with p.Q571R mutation. All Experiments were repeated three times, with similar results.

Supplementary Figure 7

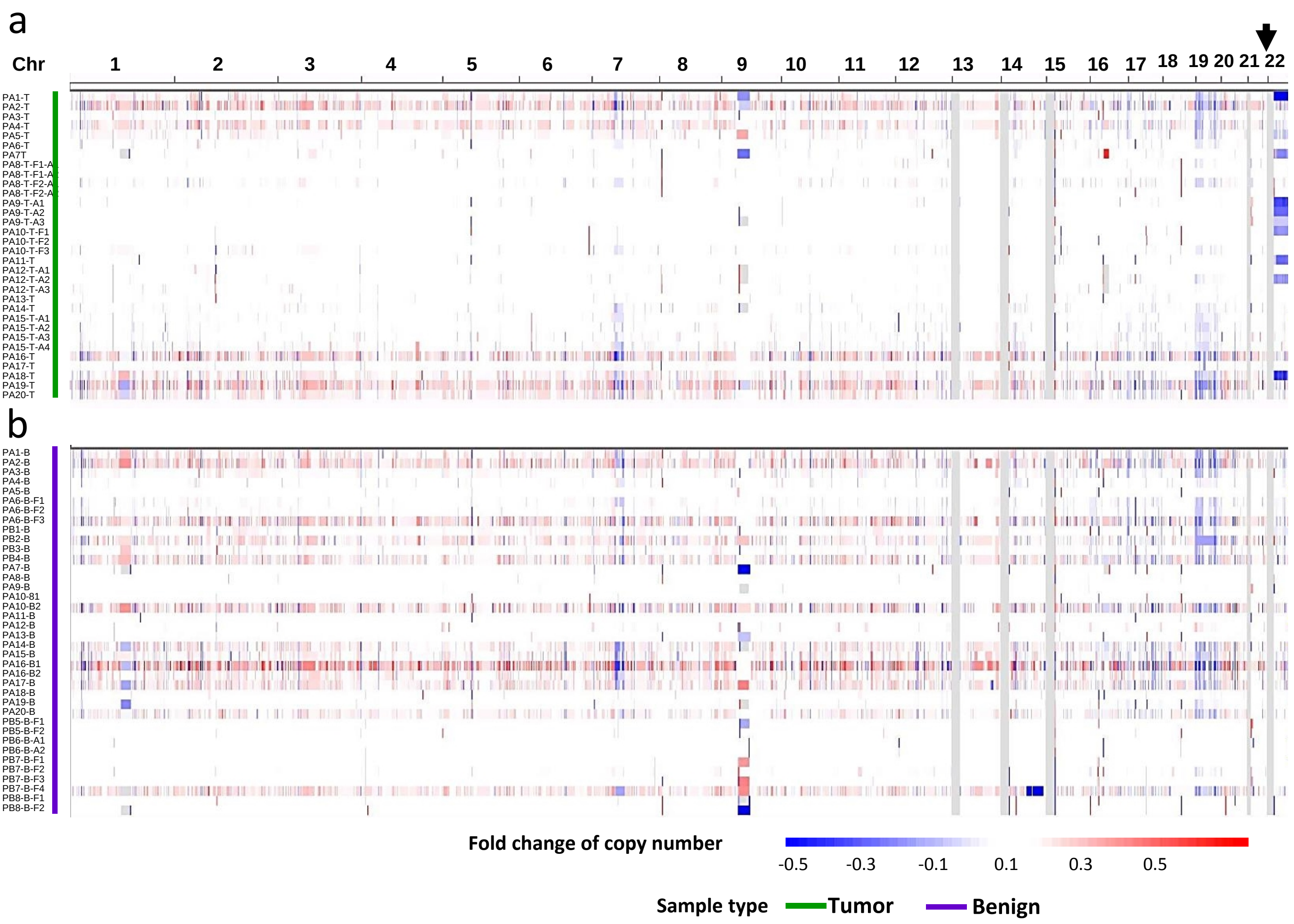

Supplementary Figure 7

Scan of the copy number variations in benign and PTC Samples

(a) PTC and (b) adenomatoid nodule samples were arranged in rows. The copy number changes in the chr (chromosome) area were represented by colors (red for gain, blue for losses and white for copy-neutral). Arrow indicated the PTC related 22q-loss.

Supplementary Figure 8

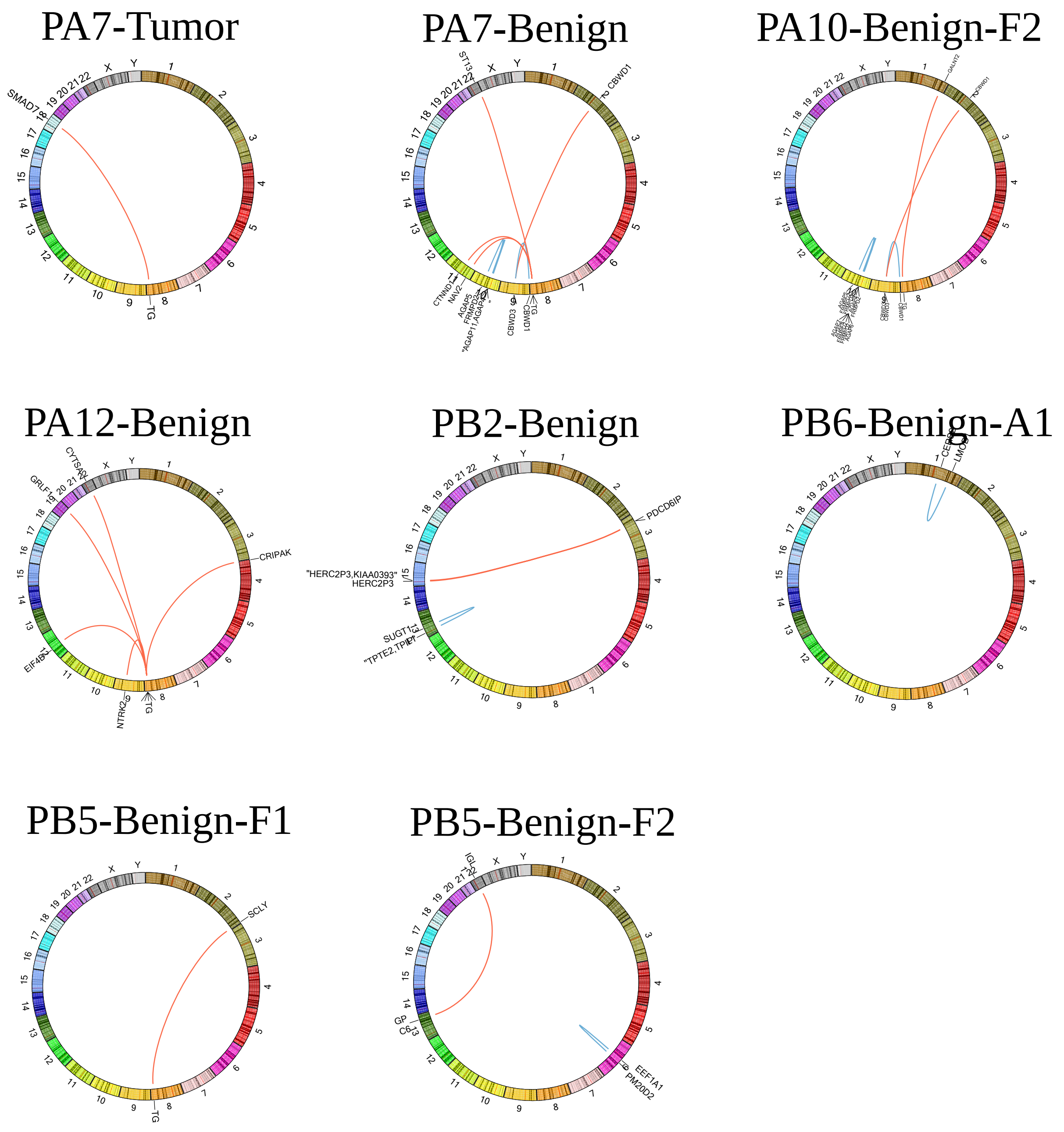

Supplementary Figure 8 Summary of gene fusions identified in our cohort
Circos plots of all the gene fusions identified in each patient by transcriptome sequencing after filtering the fusions in normal tissues. Inter-chromosomal fusion was indicated with red line, and intra-chromosomal type was indicated with blue line.

Supplementary Figure 9

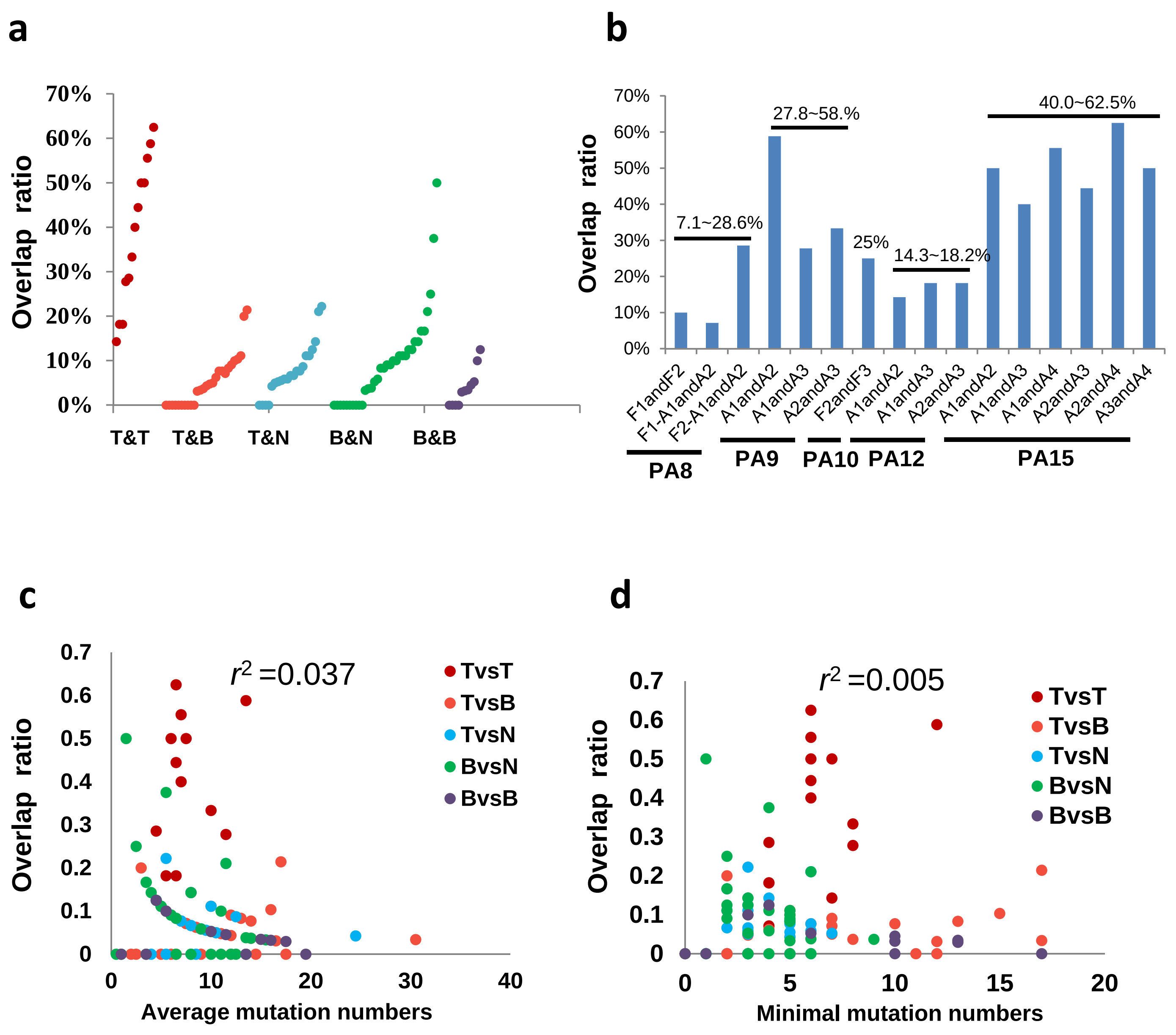

Supplementary Figure 9

Percentage of somatic mutation overlaps between sample pairs.

Overlap ratio of paired samples categorized by tissue types (a). Tumor-tumor pairs (T&T) exhibited the most overlap among others. The detailed overlap ratio of all 16 paired samples in 5 multi-sample patients were presented in (b). The overlap ratio is not correlated with either the (c) average or (d) the minimal mutation number of the paired samples.

Supplementary Figure 10

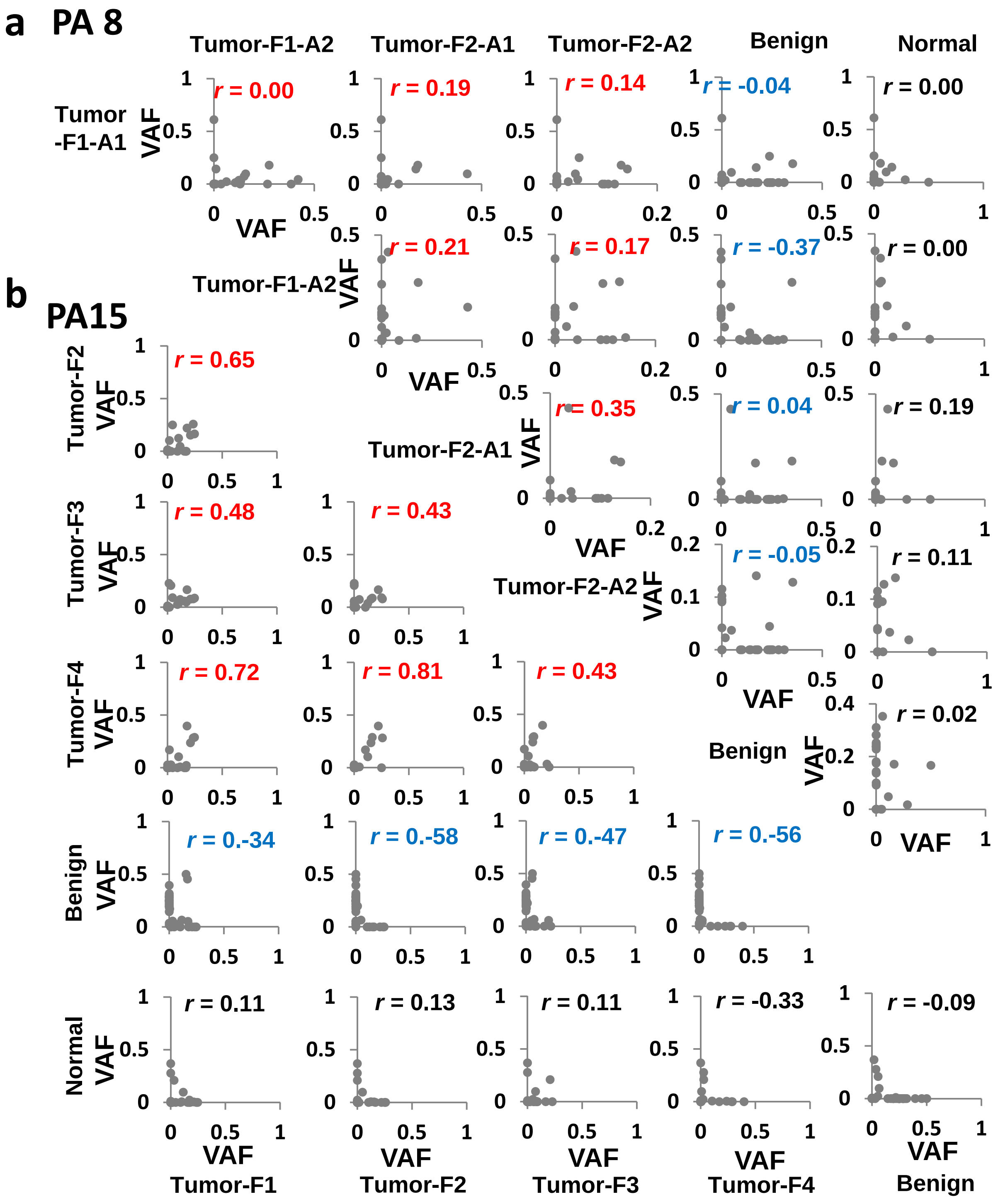

Supplementary Figure 10

Scatterplots for variation allele frequencies of paired samples in individuals PA8 and PA15.

Two patient with multi samples were showed as the example of phylogenetic unrelated (a) and distantly related (b) cases. The r value represents Pearson correlation coefficient of VAF, and it also showed consistency with the phylogenetic relationship. The color indicated the type of compared samples (red for PTC pairs, blue for PTC-benign pairs, and gray for PTC- normal pairs).

Supplementary Figure 11

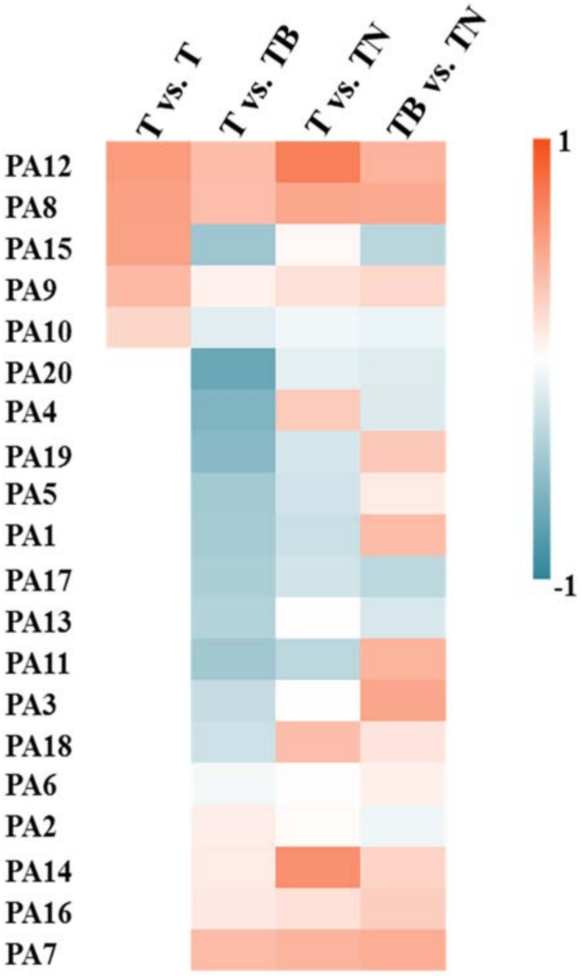

Supplementary Figure 11
Correlations of somatic mutations between paired samples in each patient. Pearson correlation of variant allele fraction (VAF) value of somatic mutations in paired samples in the 20 TB patients. The degree of the correlation was scaled by colors.

Supplementary Figure 12

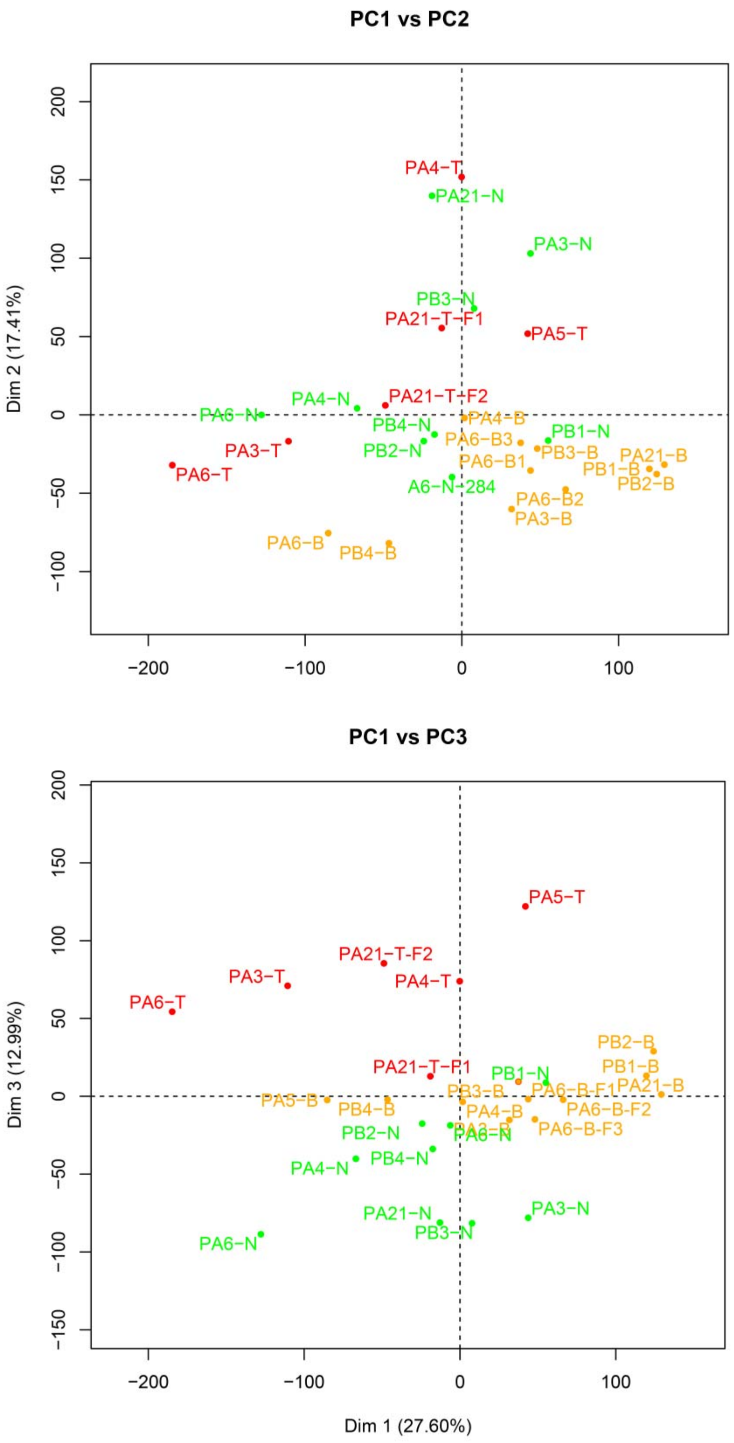

Supplementary Figure 12 The PCA of 26 samples.
Only the expressed transcripts (21,441 with the FPKM mapped reads≥1 in at least one sample) were used for analysis. Types of samples were differentiated by colors (red for PTC, orange for adenomatoid nodules and green for normal samples).

Supplementary Figure 13

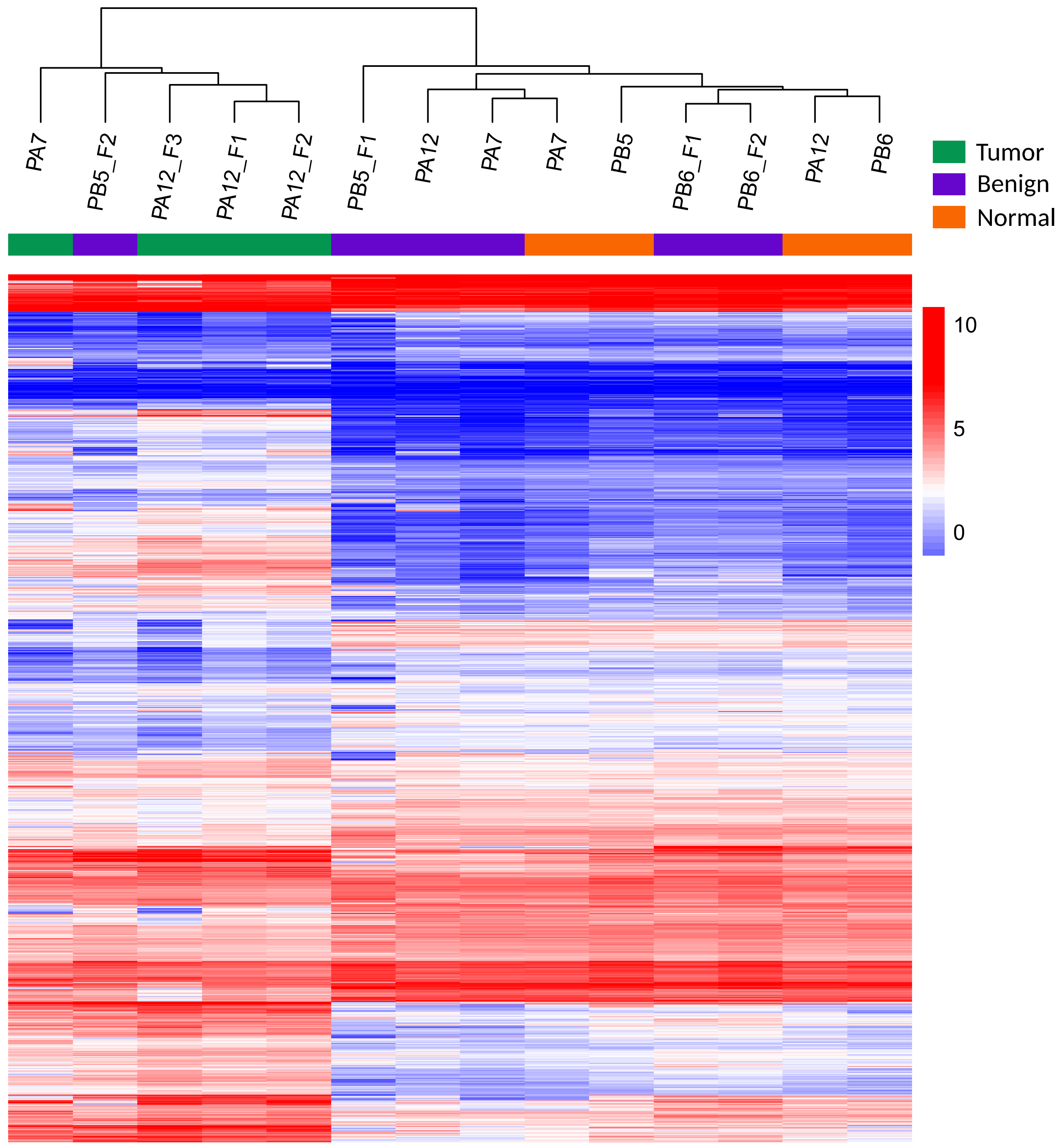

Supplementary Figure 13
The gene expression properties in an additional 14 samples from 4 patients. Hierarchical clustering analyses of whole-genome expression profiles for 14 samples in set 2. The same gene list in Fig. 5a were used. PTCs and BTNs were segregated in independent branches with the only exception of PB5.

Supplementary Figure 14

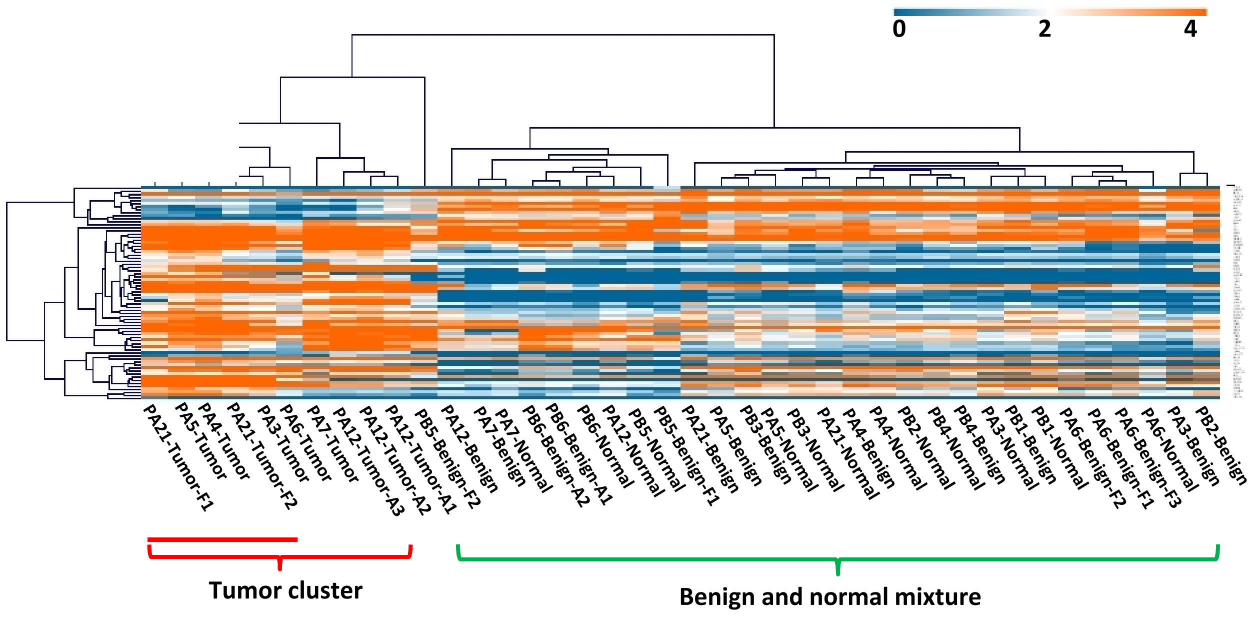

Supplementary Figure 14 The BRAF-RAS signature.
Hierarchical clustering analyses of the 71 genes set related to distinguishes BRAFV600E mutation from RAS mutation). PTCs and BTNs were segregated in independent branches with the only exception of PB5.

Supplementary Figure 15

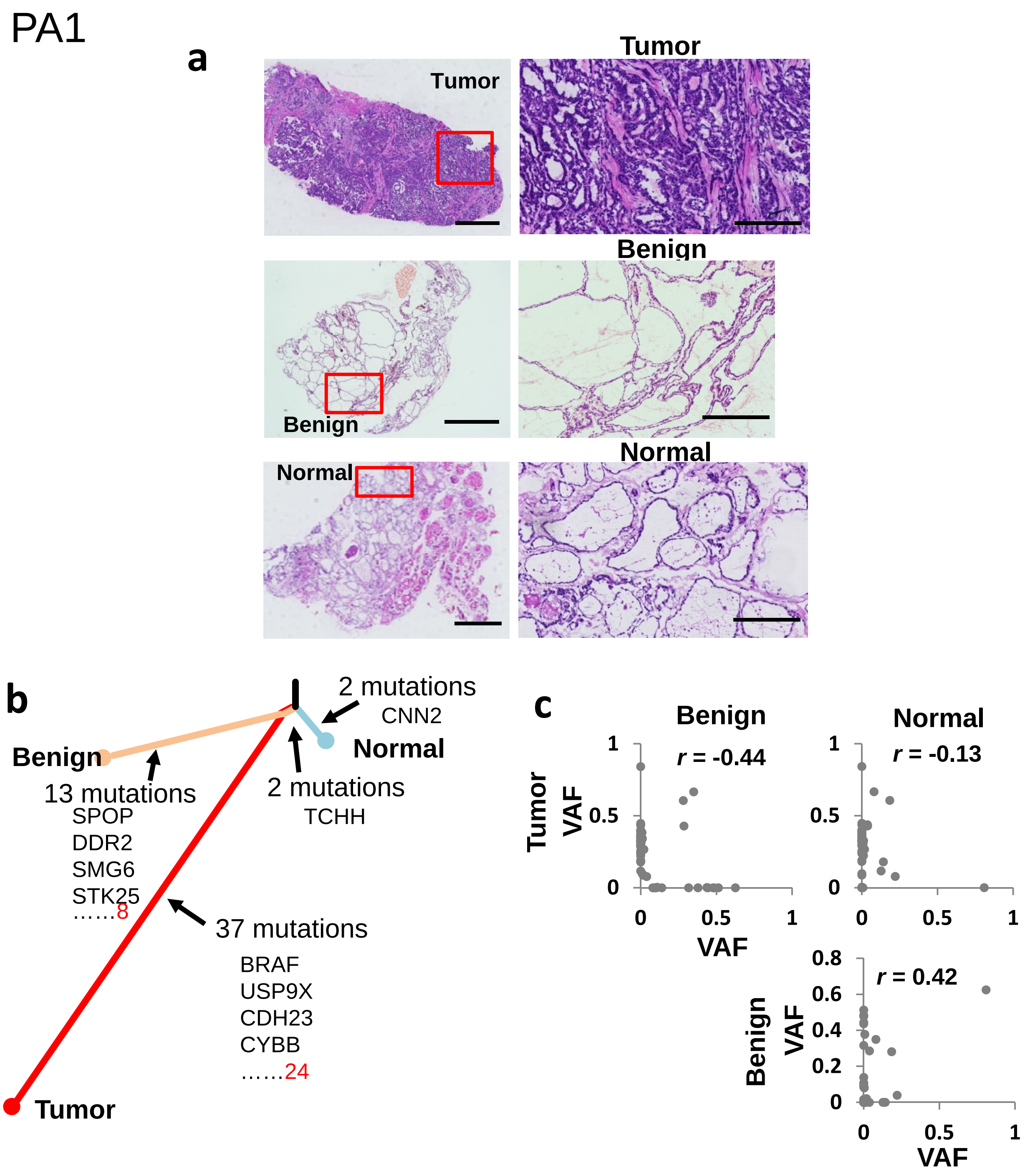

Supplementary Figure 15

The HE staining and phylogenetic relationships of samples in PA1.

(a) HE staining. The histological characters of the entire slide were showed in the left panel. The sampling region for sequencing was indicated by red box, with the enlarged photo shown in the right panel.

(b) Phylogenetic tree. The number of mutated genes are indicated near the line, and genes with nonsynonymous mutations were listed (only four were listed and the red number was count of genes with nonsynonymous mutations if *n*>4).

(c) Scatterplots for mutation allele frequencies of paired samples in each patient; The r value represents Pearson correlation coefficient.

Supplementary Figure 16

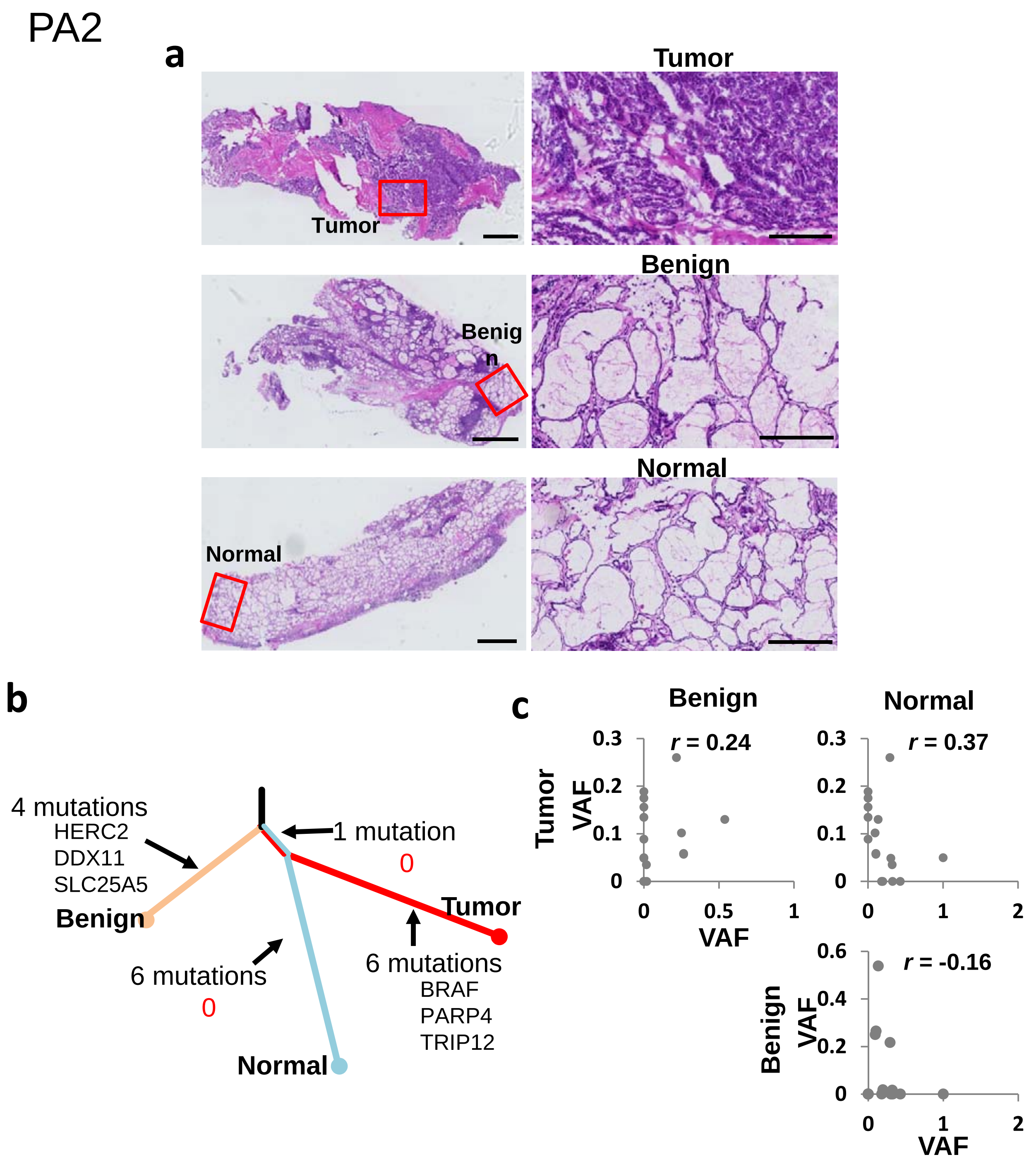

Supplementary Figure 16

The HE staining and phylogenetic relationships of samples in PA2.

(a) HE staining. The histological characters of the entire slide were showed in the left panel. The sampling region for sequencing was indicated by red box, with the enlarged photo shown in the right panel.

(b) Phylogenetic tree. The number of mutated genes are indicated near the line, and genes with nonsynonymous mutations were listed (only four were listed and the red number was count of genes with nonsynonymous mutations if *n*>4).

(c) Scatterplots for mutation allele frequencies of paired samples in each patient; The r value represents Pearson correlation coefficient.

Supplementary Figure 17

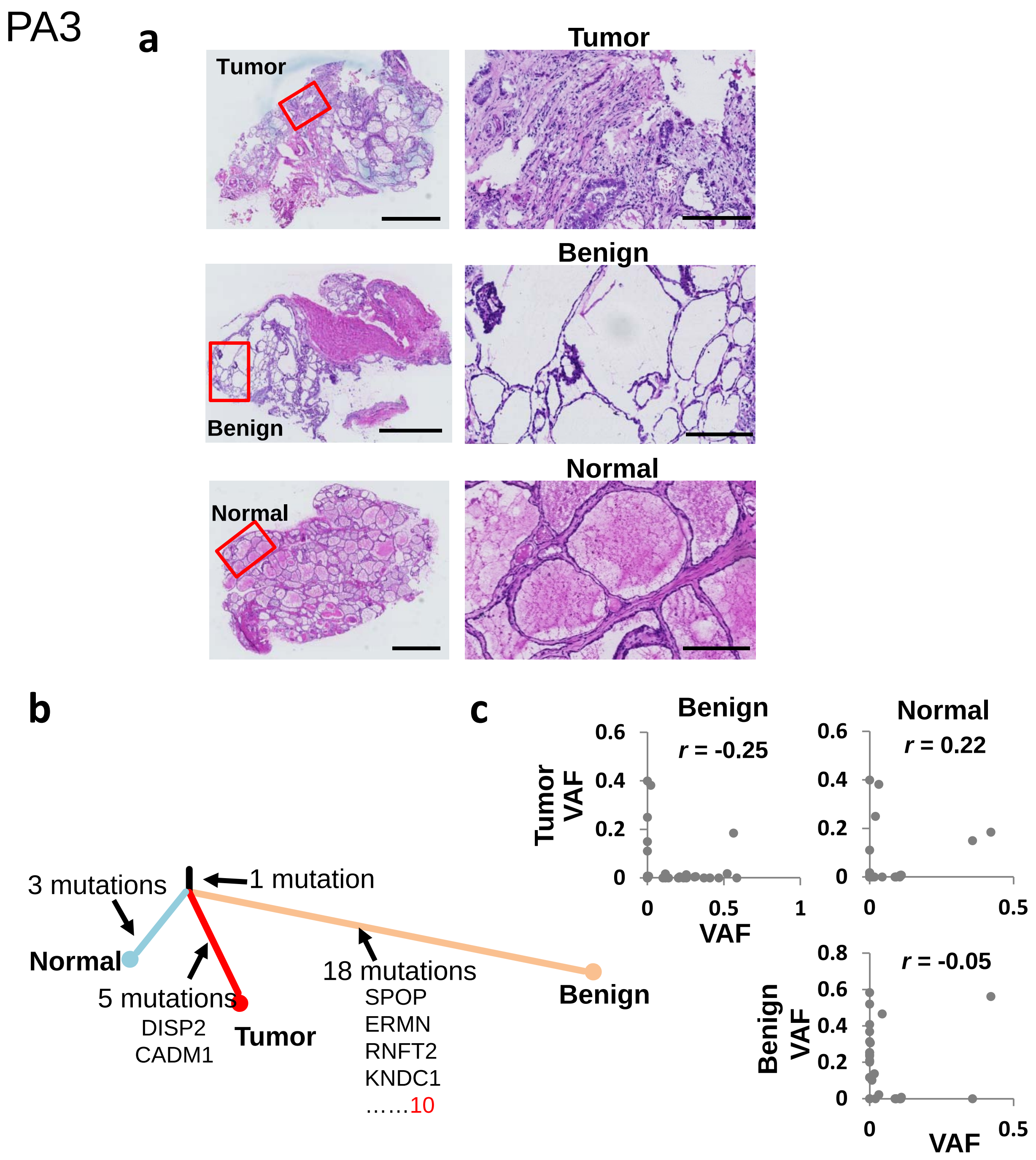

Supplementary Figure 17

The HE staining and phylogenetic relationships of samples in PA3.

(a) HE staining. The histological characters of the entire slide were showed in the left panel. The sampling region for sequencing was indicated by red box, with the enlarged photo shown in the right panel.

(b) Phylogenetic tree. The number of mutated genes are indicated near the line, and genes with nonsynonymous mutations were listed (only four were listed and the red number was count of genes with nonsynonymous mutations if *n*>4).

(c) Scatterplots for mutation allele frequencies of paired samples in each patient; The r value represents Pearson correlation coefficient.

Supplementary Figure 18

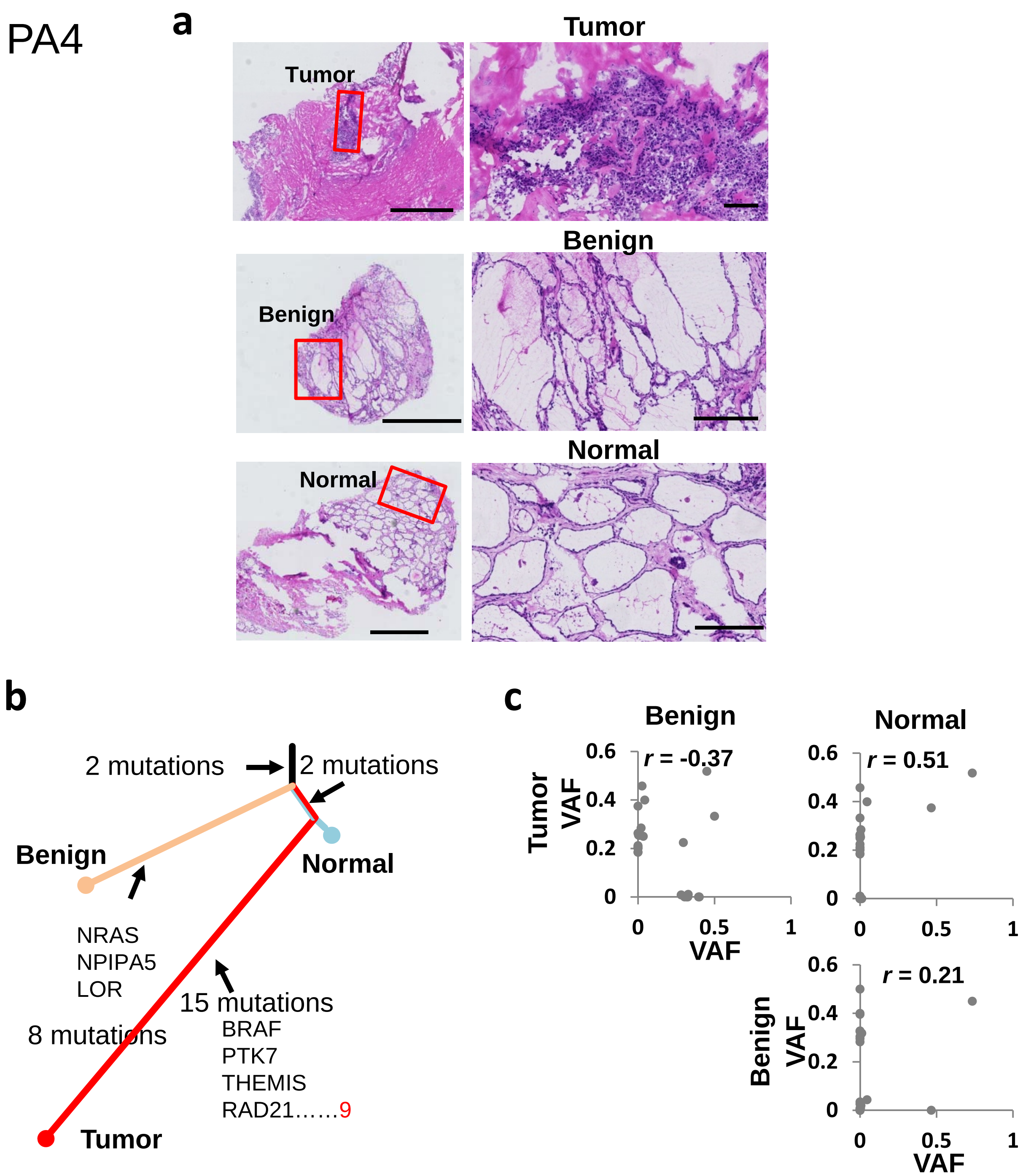

Supplementary Figure 18

The HE staining and phylogenetic relationships of samples in PA4.

(a) HE staining. The histological characters of the entire slide were showed in the left panel. The sampling region for sequencing was indicated by red box, with the enlarged photo shown in the right panel.

(b) Phylogenetic tree. The number of mutated genes are indicated near the line, and genes with nonsynonymous mutations were listed (only four were listed and the red number was count of genes with nonsynonymous mutations if *n*>4).

(c) Scatterplots for mutation allele frequencies of paired samples in each patient; The r value represents Pearson correlation coefficient.

Supplementary Figure 19

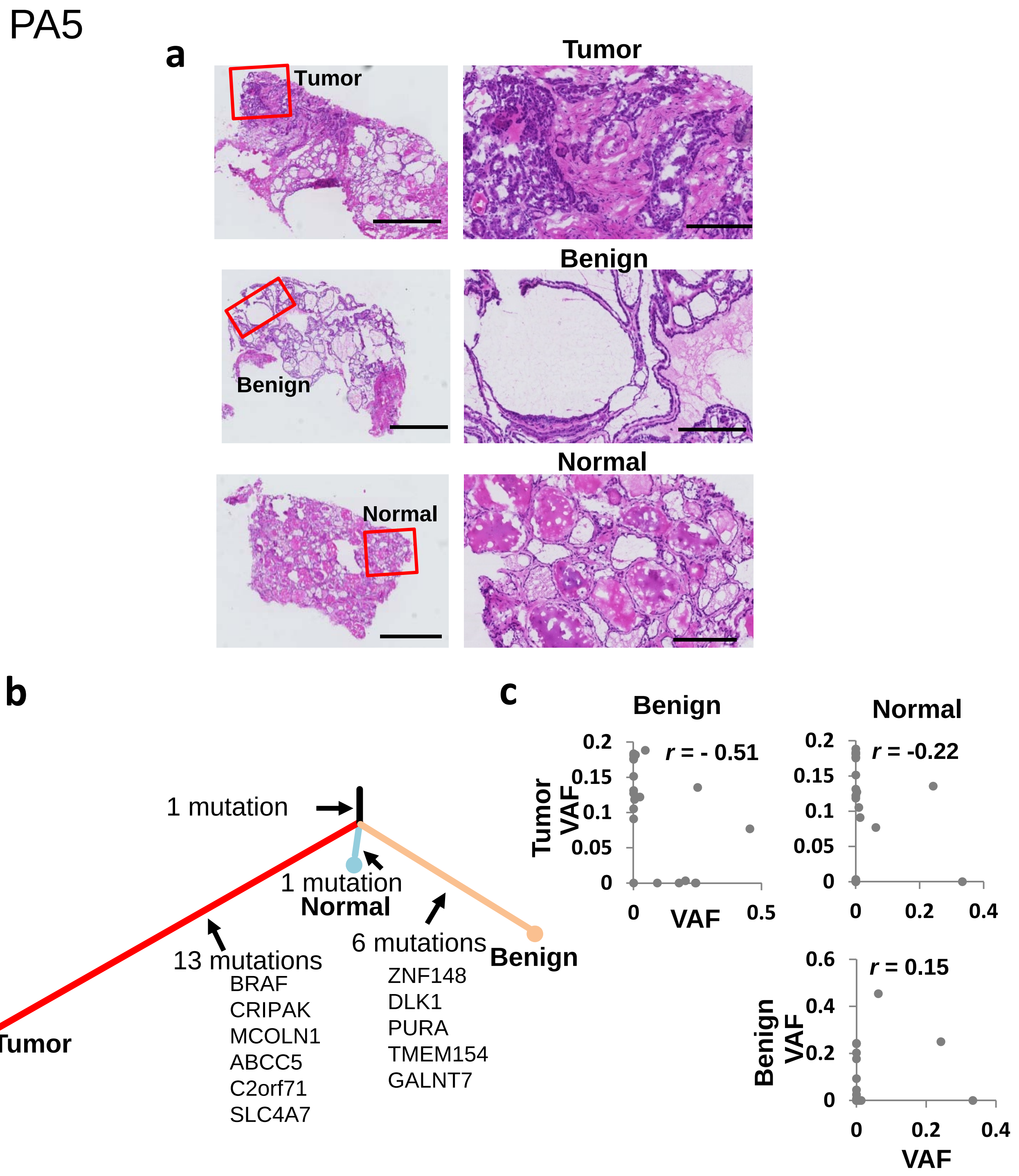

Supplementary Figure 19

The HE staining and phylogenetic relationships of samples in PA5.

(a) HE staining. The histological characters of the entire slide were showed in the left panel. The sampling region for sequencing was indicated by red box, with the enlarged photo shown in the right panel.

(b) Phylogenetic tree. The number of mutated genes are indicated near the line, and genes with nonsynonymous mutations were listed (only four were listed and the red number was count of genes with nonsynonymous mutations if *n*>4).

(c) Scatterplots for mutation allele frequencies of paired samples in each patient; The r value represents Pearson correlation coefficient.

Supplementary Figure 20

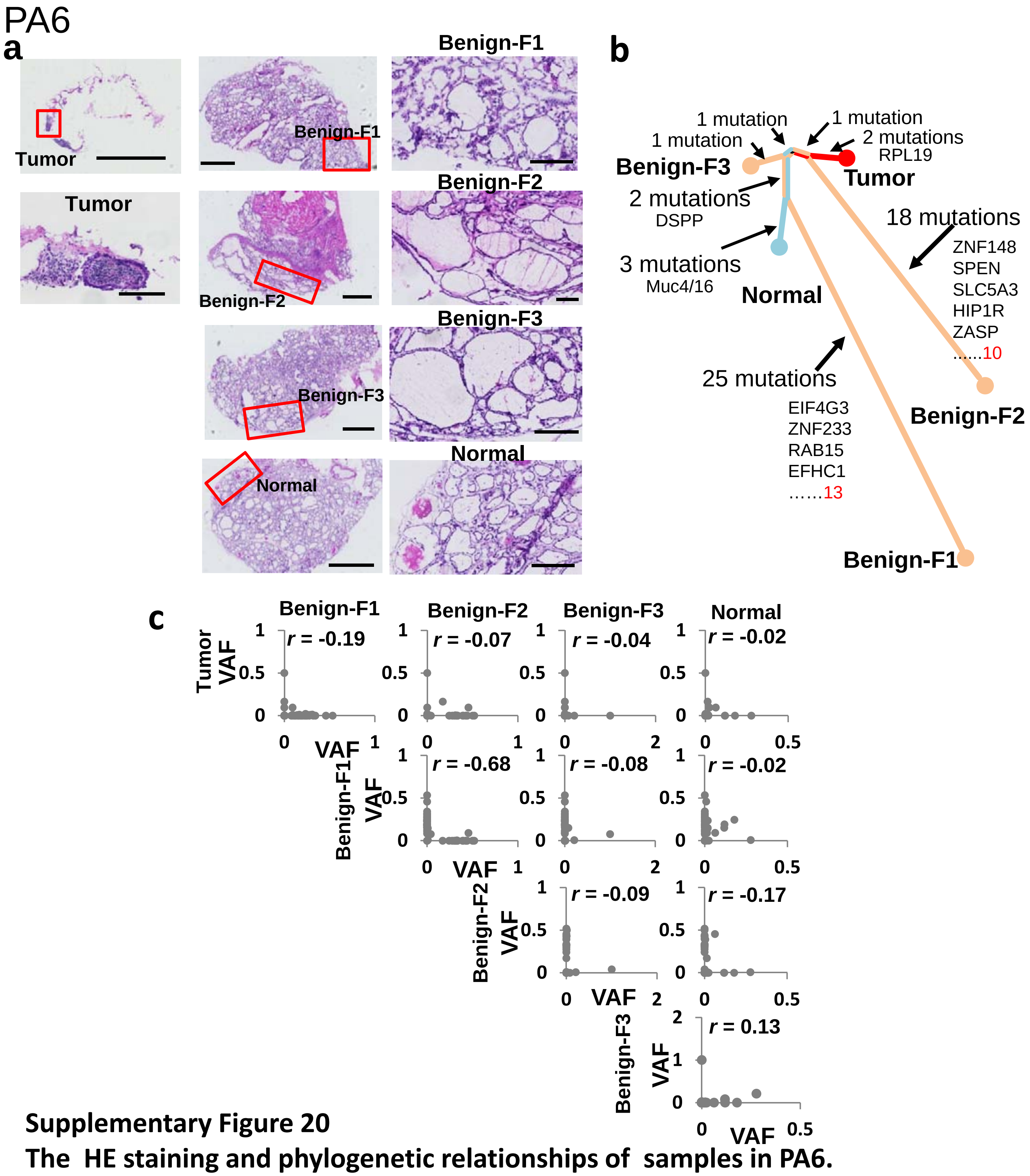

Supplementary Figure 20

The HE staining and phylogenetic relationships of samples in PA6.

(a) HE staining. The histological characters of the entire slide were showed in the left panel. The sampling region for sequencing was indicated by red box, with the enlarged photo shown in the right panel. F, foci.

(b) Phylogenetic tree. The number of mutated genes are indicated near the line, and genes with nonsynonymous mutations were listed (only four were listed and the red number was count of genes with nonsynonymous mutations if *n*>4).

(c) Scatterplots for mutation allele frequencies of paired samples in each patient; The r value represents Pearson correlation coefficient.

Supplementary Figure 21

PA7

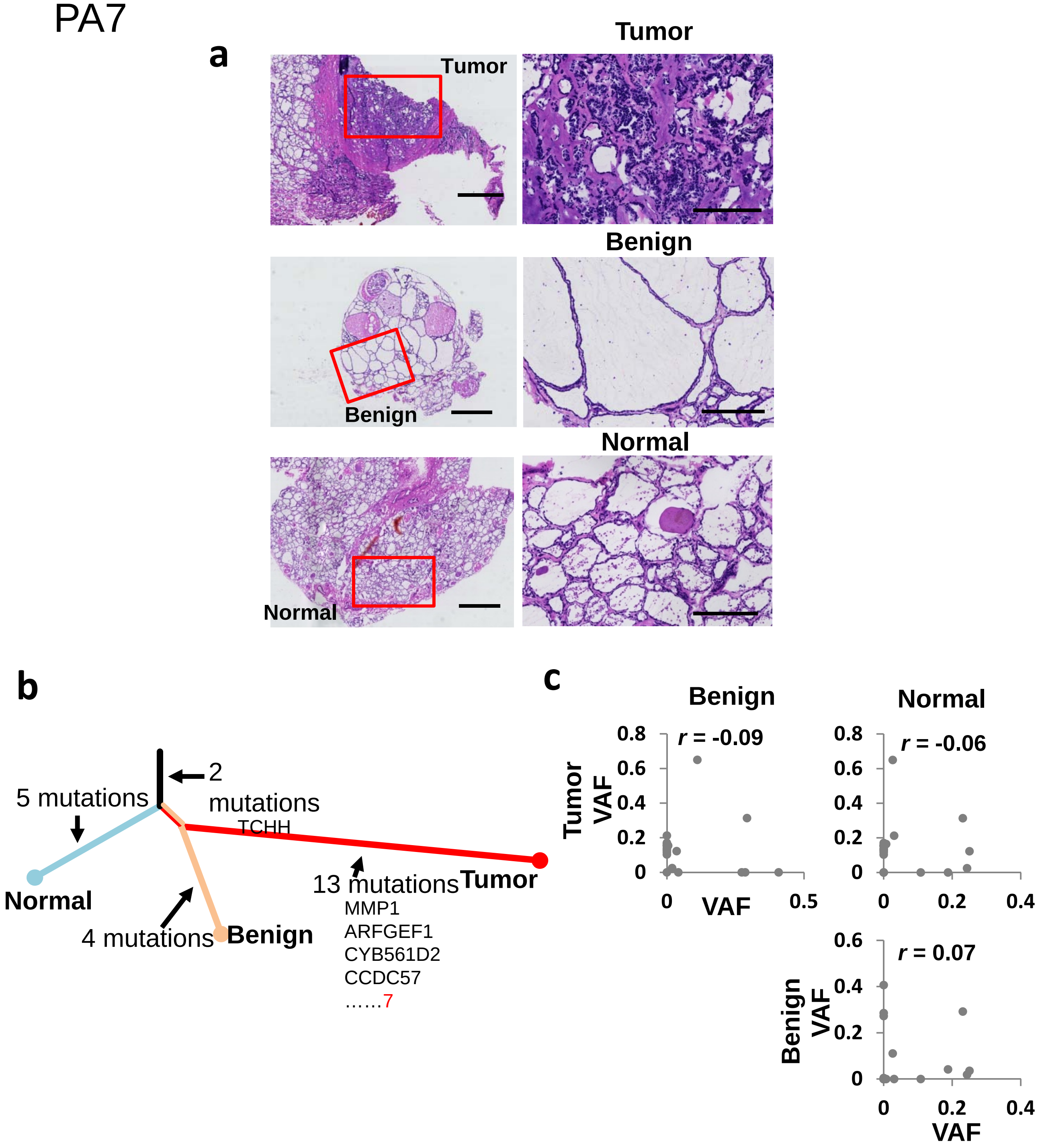

Supplementary Figure 21

The HE staining and phylogenetic relationships of samples in PA7.

(a) HE staining. The histological characters of the entire slide were showed in the left panel. The sampling region for sequencing was indicated by red box, with the enlarged photo shown in the right panel.

(b) Phylogenetic tree. The number of mutated genes are indicated near the line, and genes with nonsynonymous mutations were listed (only four were listed and the red number was count of genes with nonsynonymous mutations if *n*>4).

(c) Scatterplots for mutation allele frequencies of paired samples in each patient; The r value represents Pearson correlation coefficient.

Supplementary Figure 22

PA9

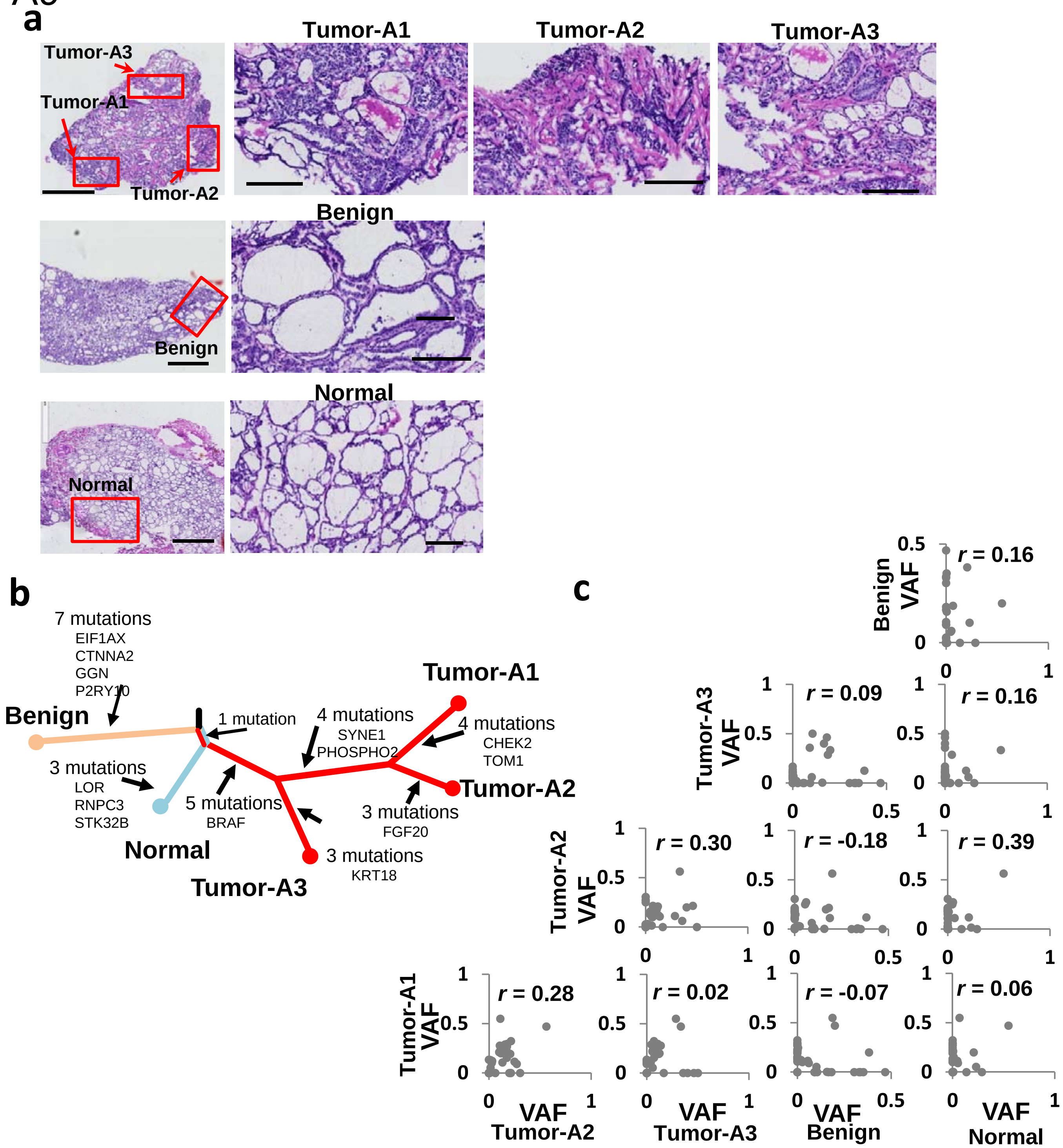

Supplementary Figure 22

The HE staining and phylogenetic relationships of samples in PA9.

(a) HE staining. The histological characters of the entire slide were showed in the left panel. The sampling region for sequencing was indicated by red box, with the enlarged photo shown in the right panel. A, area.

(b) Phylogenetic tree. The number of mutated genes are indicated near the line, and genes with nonsynonymous mutations were listed (only four were listed and the red number was count of genes with nonsynonymous mutations if *n*>4).

(c) Scatterplots for mutation allele frequencies of paired samples in each patient; The r value represents Pearson correlation coefficient.

Supplementary Figure 23

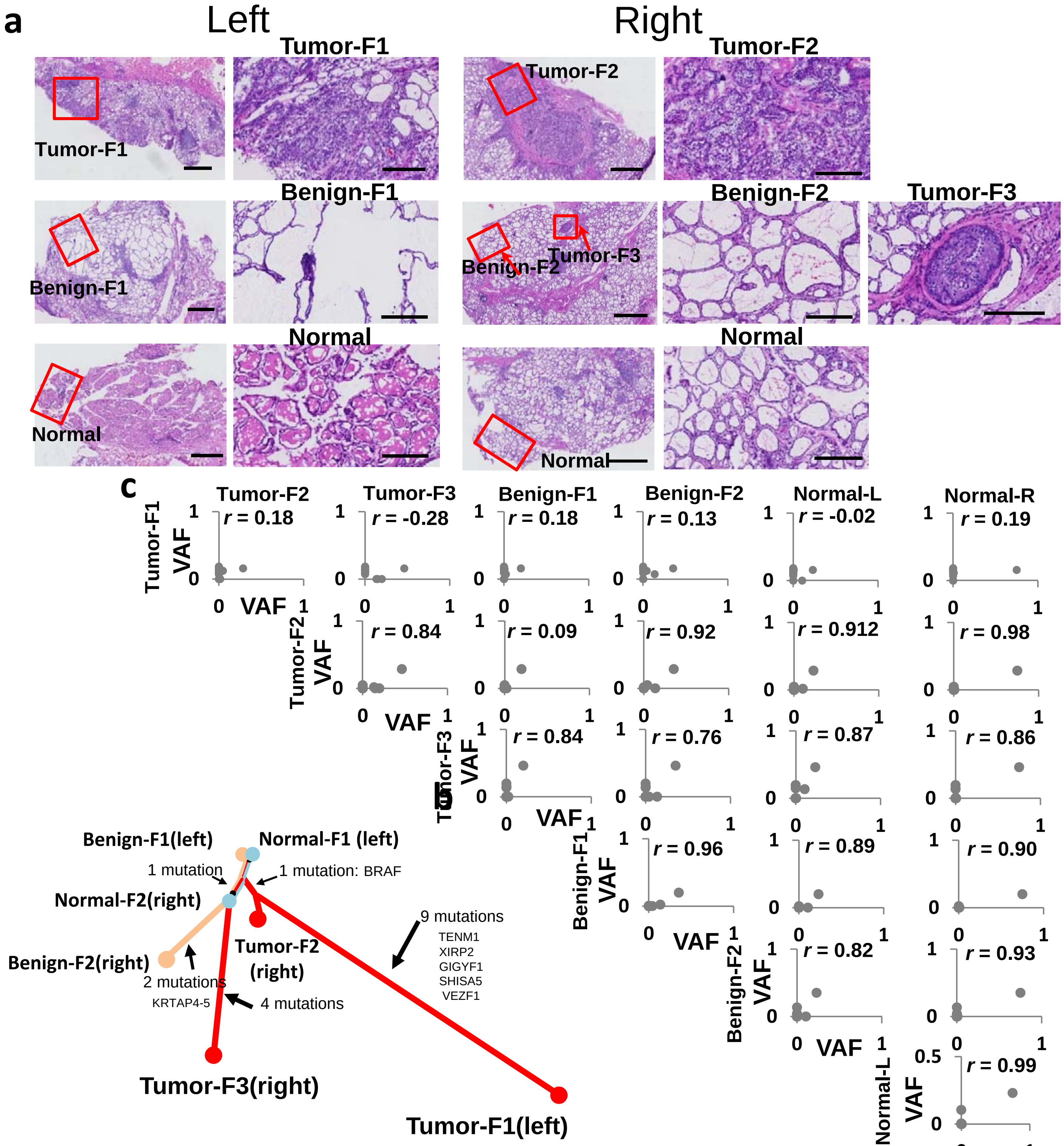

Supplementary Figure 23

The HE staining and phylogenetic relationships of samples in PA10

(a) HE staining. The histological characters of the entire slide were showed in the left panel. The sampling region for sequencing was indicated by red box, with the enlarged photo shown in the right panel. F, foci.

(b) Phylogenetic tree. The number of mutated genes are indicated near the line, and genes with nonsynonymous mutations were listed (only four were listed and the red number was count of genes with nonsynonymous mutations if *n*>4).

(c) Scatterplots for mutation allele frequencies of paired samples in each patient; The r value represents Pearson correlation coefficient.

Supplementary Figure 24

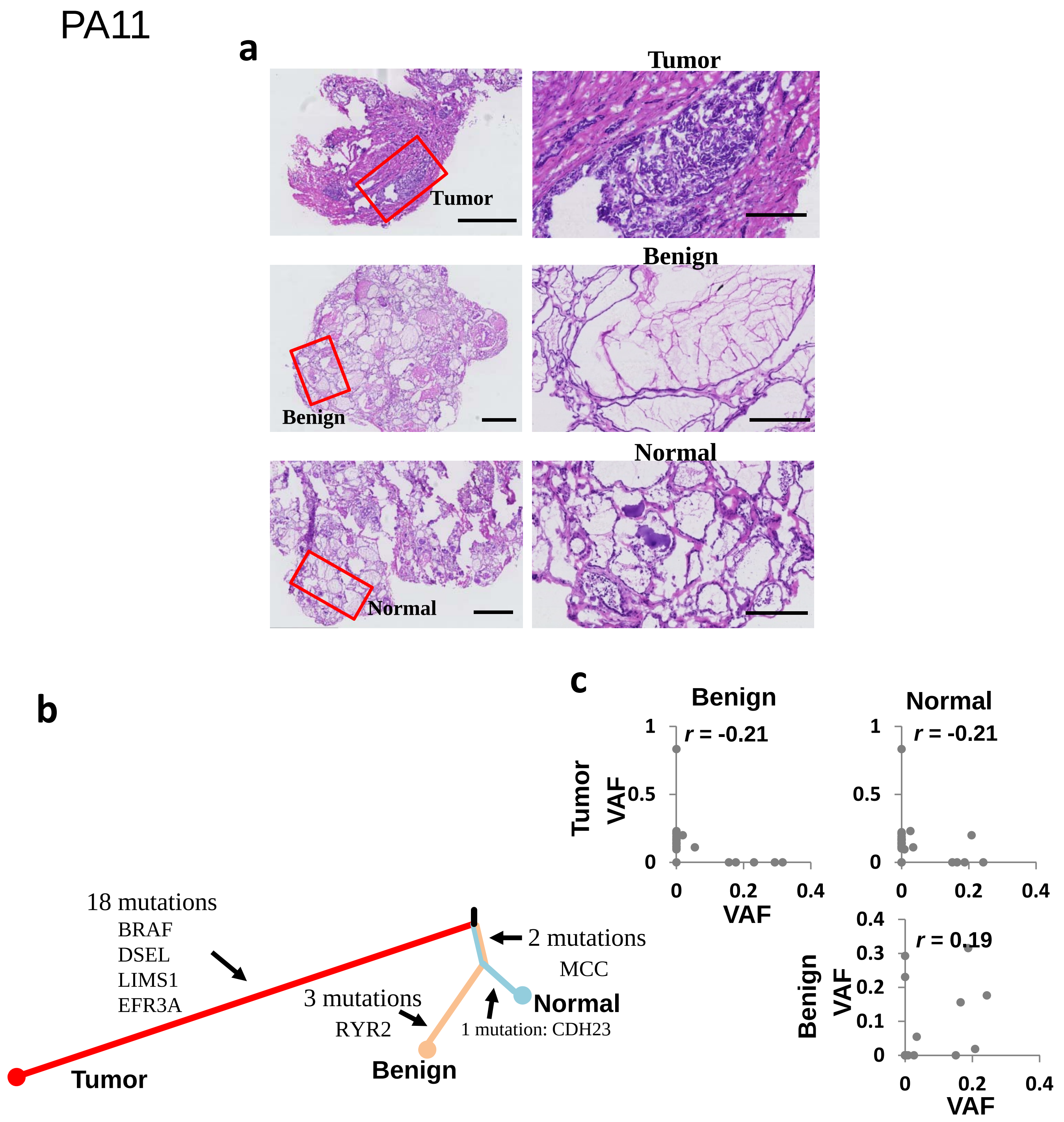

Supplementary Figure 24

The HE staining and phylogenetic relationships of samples in PA11.

(a) HE staining. The histological characters of the entire slide were showed in the left panel. The sampling region for sequencing was indicated by red box, with the enlarged photo shown in the right panel.

(b) Phylogenetic tree. The number of mutated genes are indicated near the line, and genes with nonsynonymous mutations were listed (only four were listed and the red number was count of genes with nonsynonymous mutations if n>4).

(c) Scatterplots for mutation allele frequencies of paired samples in each patient; The r value represents Pearson correlation coefficient.

Supplementary Figure 25

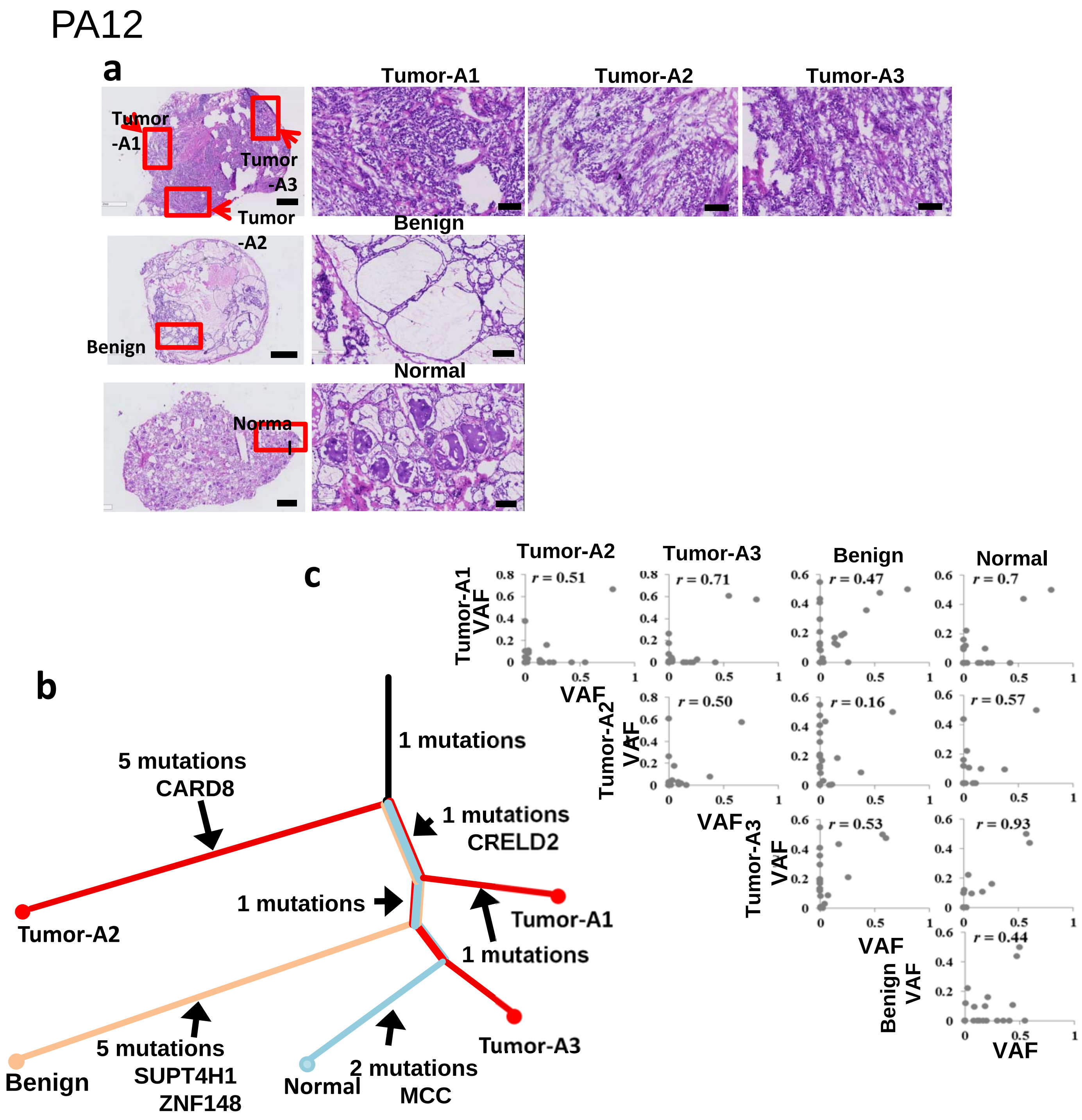

Supplementary Figure 25

The HE staining and phylogenetic relationships of samples in PA12

(a) HE staining. The histological characters of the entire slide were showed in the left panel. The sampling region for sequencing was indicated by red box, with the enlarged photo shown in the right panel. A, area.

(b) Phylogenetic tree. The number of mutated genes are indicated near the line, and genes with nonsynonymous mutations were listed (only four were listed and the red number was count of genes with nonsynonymous mutations if *n*>4).

(c) Scatterplots for mutation allele frequencies of paired samples in each patient; The r value represents Pearson correlation coefficient.

Supplementary Figure 26

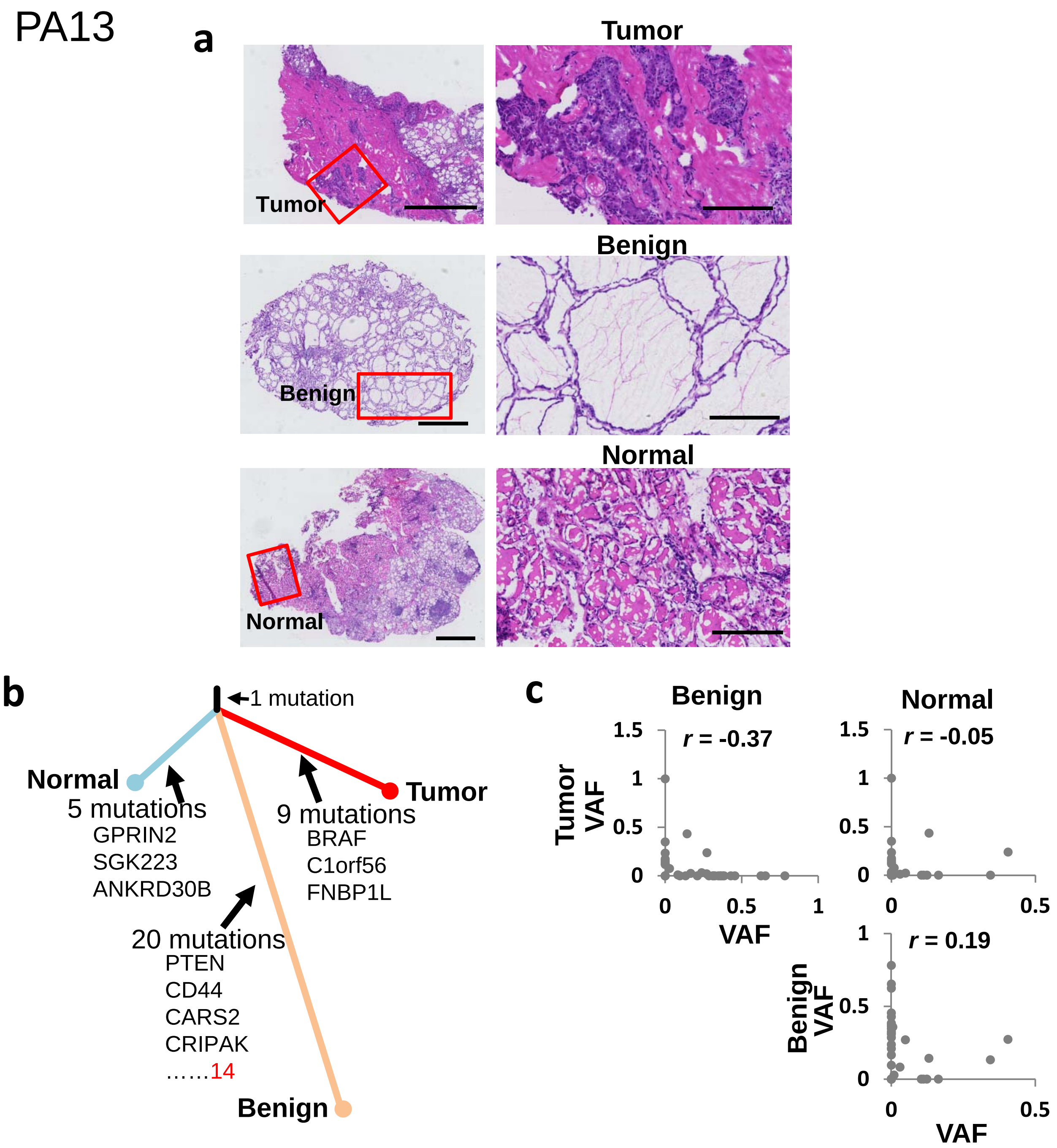

Supplementary Figure 26

The HE staining and phylogenetic relationships of samples in PA13.

(a) HE staining. The histological characters of the entire slide were showed in the left panel. The sampling region for sequencing was indicated by red box, with the enlarged photo shown in the right panel.

(b) Phylogenetic tree. The number of mutated genes are indicated near the line, and genes with nonsynonymous mutations were listed (only four were listed and the red number was count of genes with nonsynonymous mutations if n>4).

(c) Scatterplots for mutation allele frequencies of paired samples in each patient; The r value represents Pearson correlation coefficient.

Supplementary Figure 27

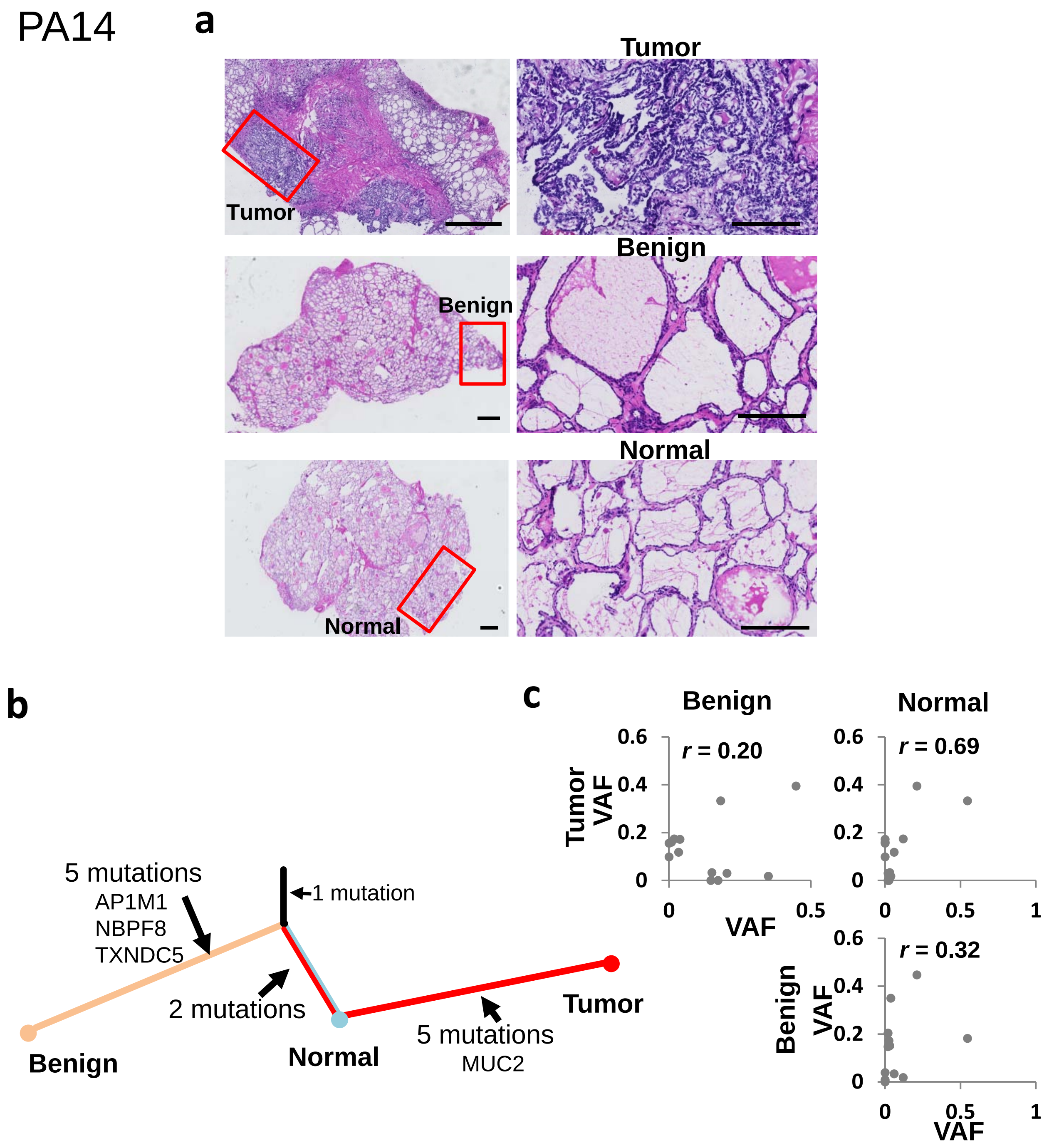

Supplementary Figure 27

The HE staining and phylogenetic relationships of samples in PA14.

(a) HE staining. The histological characters of the entire slide were showed in the left panel. The sampling region for sequencing was indicated by red box, with the enlarged photo shown in the right panel.

(b) Phylogenetic tree. The number of mutated genes are indicated near the line, and genes with nonsynonymous mutations were listed (only four were listed and the red number was count of genes with nonsynonymous mutations if n>4).

(c) Scatterplots for mutation allele frequencies of paired samples in each patient; The r value represents Pearson correlation coefficient.

Supplementary Figure 28

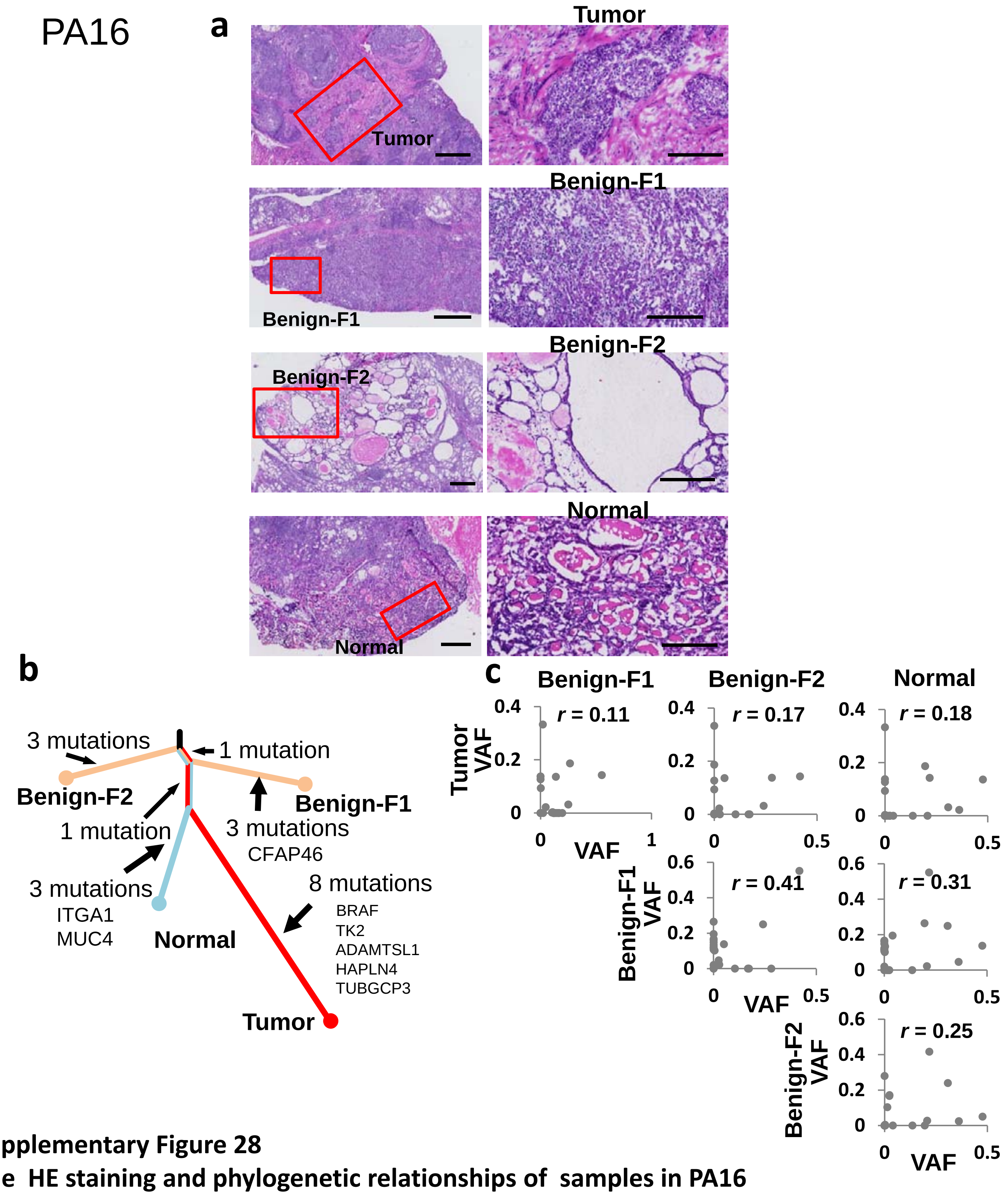

Supplementary Figure 28

The HE staining and phylogenetic relationships of samples in PA16

(a) HE staining. The histological characters of the entire slide were showed in the left panel. The sampling region for sequencing was indicated by red box, with the enlarged photo shown in the right panel. F, foci.

(b) Phylogenetic tree. The number of mutated genes are indicated near the line, and genes with nonsynonymous mutations were listed (only four were listed and the red number was count of genes with nonsynonymous mutations if *n*>4).

(c) Scatterplots for mutation allele frequencies of paired samples in each patient; The r value represents Pearson correlation coefficient.

Supplementary Figure 29

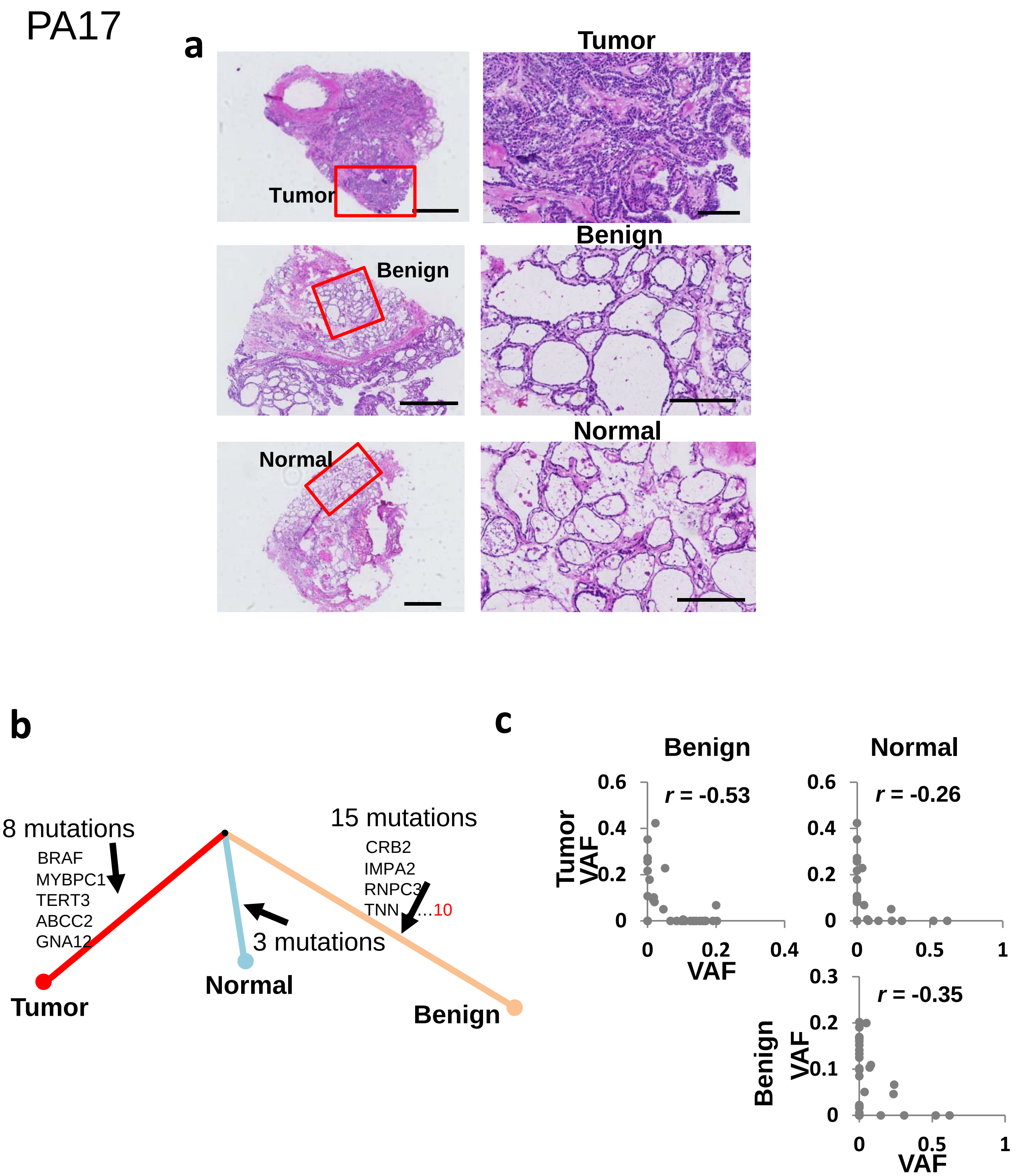

Supplementary Figure 29

The HE staining and phylogenetic relationships of samples in PA17.

(a) HE staining. The histological characters of the entire slide were showed in the left panel. The sampling region for sequencing was indicated by red box, with the enlarged photo shown in the right panel.

(b) Phylogenetic tree. The number of mutated genes are indicated near the line, and genes with nonsynonymous mutations were listed (only four were listed and the red number was count of genes with nonsynonymous mutations if *n*>4).

(c) Scatterplots for mutation allele frequencies of paired samples in each patient; The r value represents Pearson correlation coefficient.

Supplementary Figure 30

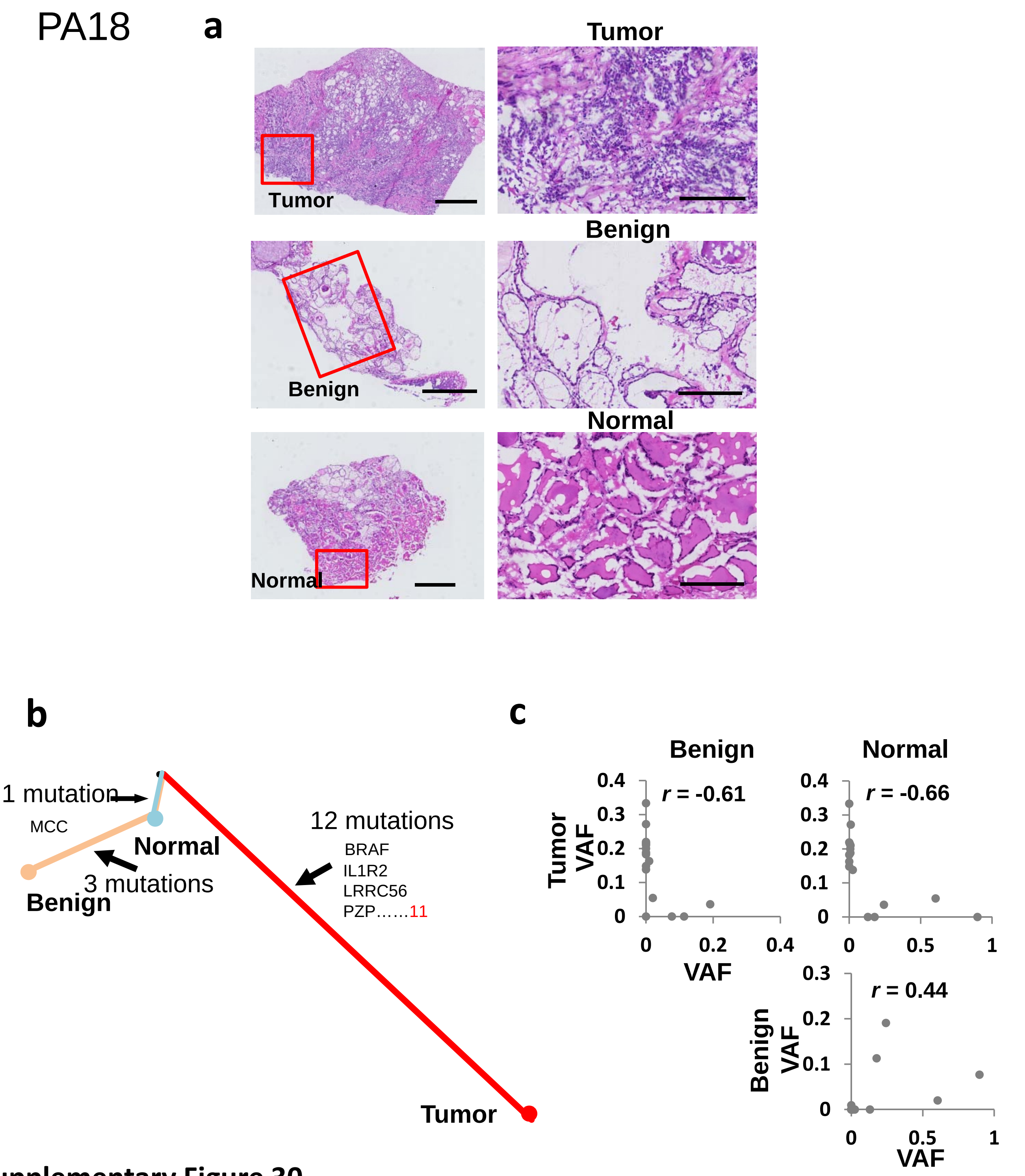

Supplementary Figure 30

The HE staining and phylogenetic relationships of samples in PA18.

(a) HE staining. The histological characters of the entire slide were showed in the left panel. The sampling region for sequencing was indicated by red box, with the enlarged photo shown in the right panel.

(b) Phylogenetic tree. The number of mutated genes are indicated near the line, and genes with nonsynonymous mutations were listed (only four were listed and the red number was count of genes with nonsynonymous mutations if n>4).

(c) Scatterplots for mutation allele frequencies of paired samples in each patient; The r value represents Pearson correlation coefficient.

Supplementary Figure 31

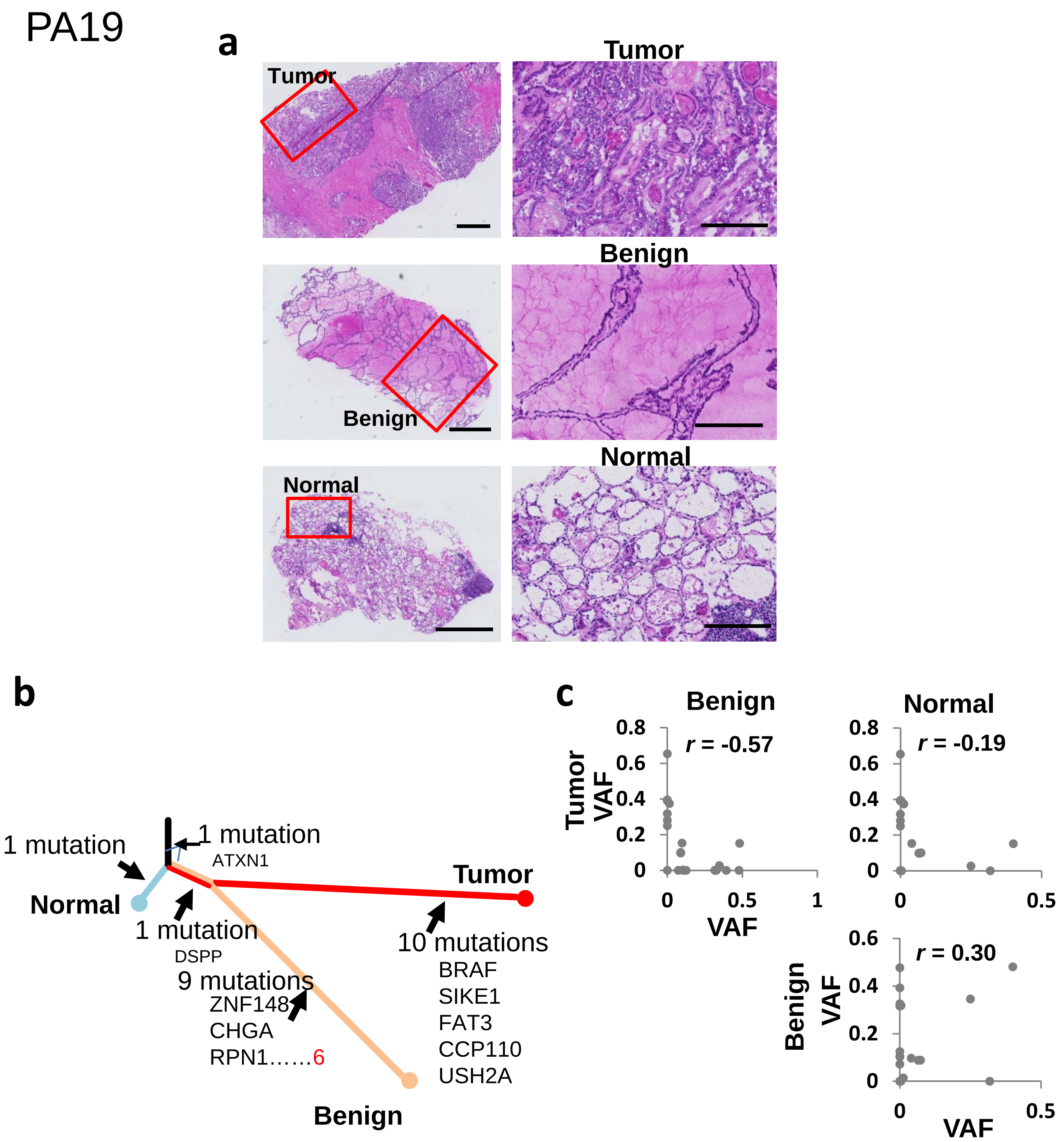

Supplementary Figure 31

The HE staining and phylogenetic relationships of samples in PA19.

(a) HE staining. The histological characters of the entire slide were showed in the left panel. The sampling region for sequencing was indicated by red box, with the enlarged photo shown in the right panel.

(b) Phylogenetic tree. The number of mutated genes are indicated near the line, and genes with nonsynonymous mutations were listed (only four were listed and the red number was count of genes with nonsynonymous mutations if *n*>4).

(c) Scatterplots for mutation allele frequencies of paired samples in each patient; The r value represents Pearson correlation coefficient.

Supplementary Figure32

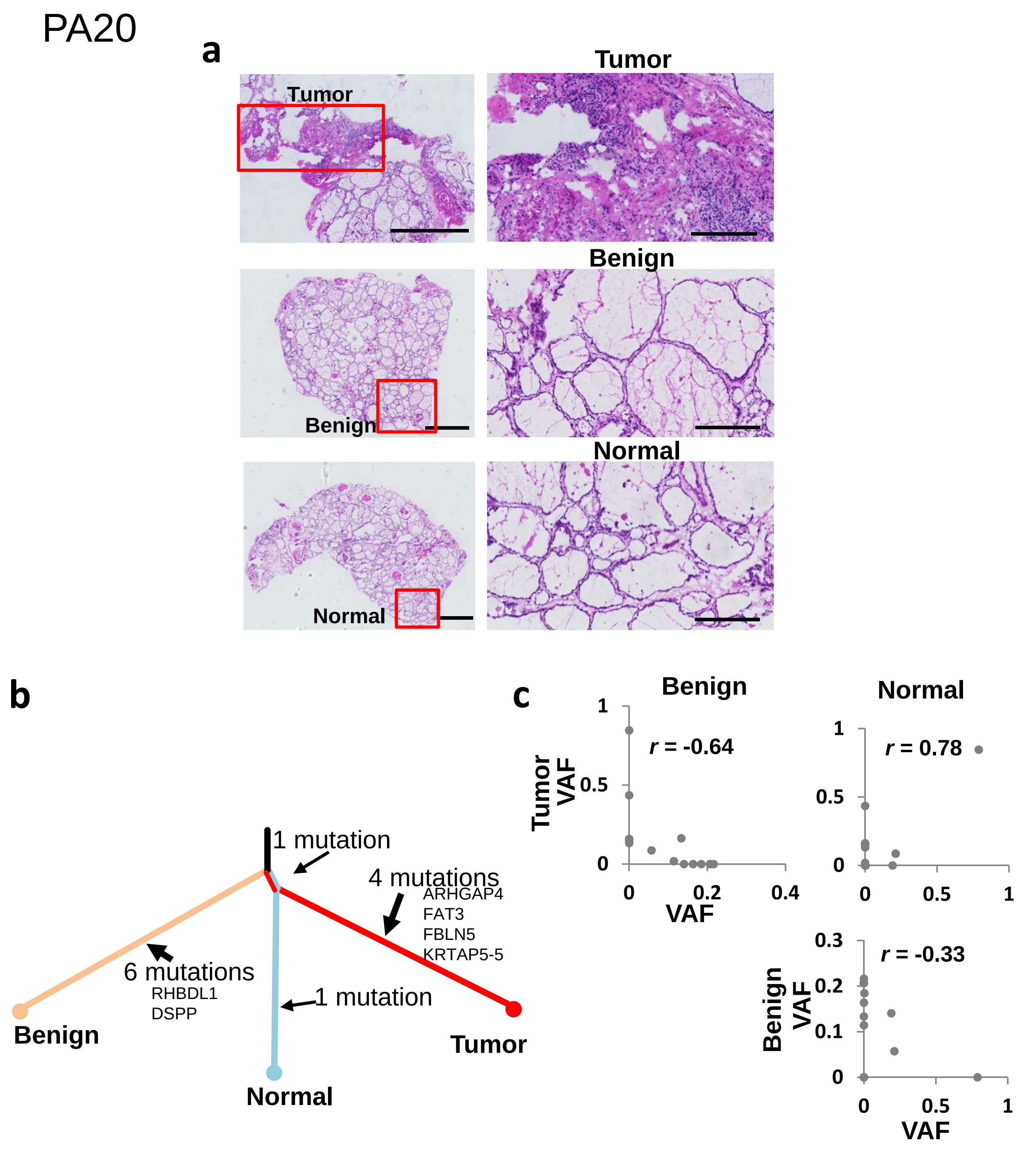

Supplementary Figure 32

The HE staining and phylogenetic relationships of samples in PA20.

(a) HE staining. The histological characters of the entire slide were showed in the left panel. The sampling region for sequencing was indicated by red box, with the enlarged photo shown in the right panel.

(b) Phylogenetic tree. The number of mutated genes are indicated near the line, and genes with nonsynonymous mutations were listed (only four were listed and the red number was count of genes with nonsynonymous mutations if *n*>4).

(c) Scatterplots for mutation allele frequencies of paired samples in each patient; The r value represents Pearson correlation coefficient.

Supplementary Table 1
Mutation frequency of SPOP, ZNF148, EZH1 in TCGA database

Tumor Type	*SPOP*	*ZNF148*	*EZH1*
**BLCA** *(Bladder Urothelial Carcinoma , N = 412)*	2 (0.49%)	3 (0.73%)	2 (0.49%)
**BRCA** *(Breast invasive carcinoma , N = 1097)*	5 (0.46%)	6 (0.55%)	3 (0.27%)
**KIRC** *(Kidney renal clear cell carcinoma , N = 536)*	0	3 (0.56%)	1 (0.19%)
**CESC** *(Cervical squamous cell carcinoma and endocervical adenocarcinoma , N = 307)*	1 (0.33%)	2 (0.65%)	4 (1.30%)
**COAD/READ** *(Colorectal adenocarcinoma, N = 632)*	2 (0.32%)	3 (0.47%)	5 (0.79%)
**DLBC** *(Lymphoid Neoplasm Diffuse Large B-cell Lymphoma, N = 48*)	1 (2.08%)	0	0
**GBM** *(Glioblastoma multiforme , N = 528)*	2 (0.38%)	3 (0.57%)	1 (0.19%)
**LGG** *(Brain Lower Grade Glioma , N = 516)*	1 (0.19%)	2 (0.39%)	1 (0.19%)
**HNSC** *(Head and Neck squamous cell carcinoma , N = 528)*	4 (0.76%)	3 (0.57%)	2 (0.38%)
**LIHC** *(Liver hepatocellular carcinoma , N = 377)*	4 (1.06%)	0	1 (0.27%)
**LUAD** *(Lung adenocarcinoma , N = 521)*	2 (0.38%)	2 (0.38%)	3 (0.58%)
**LUSC** *(Lung squamous cell carcinoma , N = 504)*	1 (0.20%)	3 (0.60%)	5 (0.99%)
**UVM** *(Uveal Melanoma , N = 80)*	2 (2.50%)	9 (11.25%)	9 (11.25%)
**OV** *(Ovarian serous cystadenocarcinoma , N = 586)*	1 (0.17%)	1 (0.17%)	4 (0.68%)
**PAAD** *(Pancreatic adenocarcinoma , N = 185)*	1 (0.54%)	1 (0.54%)	2 (1.08%)
**PCPG** *(Pheochromocytoma and Paraganglioma , N = 179)*	0	1 (0.56%)	0
**KIRP** *(Kidney renal papillary cell carcinoma , N = 291)*	0	0	1 (0.34%)
**PRAD** *(Prostate adenocarcinoma , N = 498)*	38 (7.63%)	4 (0.80%)	2 (0.40%)
**SARC** *(Sarcoma , N = 261)*	0	1 (0.38%)	0
**STAD** *(Stomach adenocarcinoma , N = 443)*	3 (0.68%)	7 (1.58%)	5 (1.13%)
**THCA** *(Thyroid carcinoma , N = 507)*	1 (0.20%)	2 (0.39%)	2 (0.39%)
**UCEC/UCS** *(Uterine Corpus Endometrial Carcinoma / Uterine Carcinosarcoma , N = 605)*	28 (4.63%)	13 (2.15%)	10 (1.65%)

Supplementary Table 2
The type of PTC-Benign genetic evolutionary relationship of 20 TB patients

Patient	Type	Phynotype
PA1	distantly related	Benign and Tumor share 2 mutations not seen in normal thyroid
PA2	unrelated	Benign and Tumor independent
PA3	relatively unrelated	Benign and Tumor independent when normal thyroid considered
PA4	relatively unrelated	Benign and Tumor independent when normal thyroid considered
PA5	relatively unrelated	Benign and Tumor independent when normal thyroid considered
PA6	distantly related	Benign-F2 and Tumor share 1 mutation not seen in normal thyroid
PA7	distantly related	Benign and Tumor share higher correlations than in normal thyroid
PA8	distantly related	Benign and Tumor share 1 mutation not seen in normal thyroid
PA9	unrelated	Benign and Tumor independent
PA10	relatively unrelated	Benign and Tumor independent when normal thyroid considered
PA11	unrelated	Benign and Tumor independent
PA12	relatively unrelated	Benign and Tumor independent when normal thyroid considered
PA13	relatively unrelated	Benign and Tumor independent when normal thyroid considered
PA14	relatively unrelated	Benign and Tumor independent when normal thyroid considered
PA15	unrelated	Benign and Tumor independent
PA16	relatively unrelated	Benign and Tumor independent when normal thyroid considered
PA17	unrelated	Benign and tumor independent
PA18	unrelated	Benign and Tumor independent
PA19	distantly related	Benign and Tumor share 1 mutation not seen in normal thyroid
PA20	unrelated	Benign and Tumor independent
